# Supplementary material for: A novel role for the peptidyl-prolyl cis-trans isomerase Cyclophilin A in DNA-repair following replication fork stalling via the MRE11-RAD50-NBS1 complex
Source: EMBO Rep. 2024 Jun 28;25(8):3432–55. doi: 10.1038/s44319-024-00184-9 (PMC11315929; doi:10.1038/s44319-024-00184-9)
Supplement: Supplementary file 1 — Appendix [file 44319_2024_184_MOESM1_ESM.pdf]

Appendix Material.

Table of Contents.

	Page No.
**Appendix Table S1.**	**2**
**Appendix Table S2.**	**3**
**Appendix Table S3.**	**4**
**Appendix Table S4.**	**5**
**Appendix Table S5.**	**6-7**
**Appendix Figure S1.**	**8**
**Appendix Figure S2.**	**9**
**Appendix Figure S3.**	**10**
**Appendix Figure S4.**	**11**
**Appendix Figure S5.**	**12**
**Appendix Figure S6.**	**13**
**Appendix Figure S7.**	**14-15**
**Appendix Figure S8.**	**16**
**Appendix Table S6:** ***Cell lines.***	**17-18**
**Appendix Table S7:** ***Antibodies.*** **Appendix Materials &**	**19**
**Appendix Table S8:** ***siRNA.***	**20**
**Appendix Table S9:** ***Vectors and Site Directed Mutagenesis (SDM).***	**21**

Protein	# Peptides	Conditions
**ANLN**	24	Unt & HU
**MASTL/GWL**	3	HU
**AURKA**	2	Unt
**PLK1**	2	Unt
**BUB1B**	3	HU
**SASS6**	3	HU
**CEP170**	2	Unt & HU
**CEP85**	2	HU
**CEP97**	2	Unt
**CENPF**	2	HU
**CHAMP1**	8	Unt & HU
**INCENP**	4	Unt & HU
**MACF1**	4	Unt
**MAPRE2**	2	Unt
**MAP1A**	2	HU
**EML4**	5	HU
**TBCB**	2	Unt & HU
**TTLL12**	2	HU
**TUBA3C**	5	HU
**KIFC1**	7	HU
**KIF23**	9	Unt & HU
**KIF11/EG5**	5	Unt & HU
**KIF14**	2	Unt

Appendix Table S1.

Spindles, kinetochore, centrosome, and microtubule ‘hits’ from CYPA-BioID. Conditions under which each individual candidate ‘hit’ was detected: Unt; untreated. HU; 1mM 18hrs.

Protein	# Peptides	Conditions
**hnRNPAB**	3	HU
**hnRNPC**	2	Unt & HU
**hnRNPDL**	2	Unt & HU
**hnRNPH1**	2	Unt & HU
**hnRNPH3**	3	Unt
**DDX8/DHX8**	2	Unt & HU
**DDX3Y**	2	Unt & HU
**DDX39B/UAP36**	2	Unt
**CWC15**	2	HU
**CPSF2**	3	Unt
**CPSF7**	3	Unt & HU
**NUDT21**	4	Unt & HU
**PHAX**	4	HU
**FUS**	2	Unt & HU
**RTF1**	3	Unt & HU
**POLR2A**	4	Unt
**MED1**	3	Unt & HU
**MED12**	3	Unt
**CTCF**	5	HU
**RBM22**	2	Unt & HU
**WDR33**	3	Unt & HU
**WDR36**	2	Unt & HU
**FMR1**	2	HU
**RPRD2**	2	Unt & HU
**ALYREF/THOC4**	2	Unt
**APOBEC3B**	2	HU
**POLDIP3**	5	Unt & HU
**ILF2**	3	Unt & HU
**ILF3**	3	Unt & HU
**TTF2**	11	HU
**SIN3A**	3	Unt & HU
**PRPF31**	3	Unt
**SNRNP70**	4	Unt
**CARS**	3	Unt & HU
**WARS**	3	Unt
**RBM22**	2	Unt & HU
**AIMP1**	2	Unt & HU
**ADAR1**	2	HU
**PRPF38B**	5	Unt & HU
**SCAF4**	2	Unt & HU
**MPHOSPH10**	3	HU
**PABPC4**	3	Unt & HU
**ZNF131**	2	HU
**ZNF148**	2	HU
**ZNF207**	3	Unt & HU
**ZNF280C**	3	HU
**ZNF609**	2	HU
**ZNF687**	2	Unt & HU

Appendix Table S2.

Spliceosome, Transcription, RNA binding and R Loop. Conditions: Unt; untreated. HU; 1mM, 18hrs.

<table>
<tr><th>Variant</th><th>PolyPhen2</th><th>MutationTaster</th></tr>
<tr><td rowspan="2">P112G</td><td>Score: 1
Prediction: “Probably Damaging”</td><td>Grantham Matrix Score: 42
Prediction: “Disease Causing”</td></tr>
<tr><th colspan="2">gnomAD</th></tr>
<tr><td>P112L</td><td colspan="2">A very rare VUS with allele frequency of 3.19×10^{-5} (1x allele detected in 31392 analysed).
Not seen as a homozygous.
PolyPhen2 Score: 1 Prediction: “Probably Damaging”</td></tr>
<tr><td>P112R</td><td colspan="2">A very rare VUS with allele frequency of 3.99×10^{-6} (1x allele detected in 250872 analysed).
Not seen as a homozygous.
PolyPhen2 Score: 1 Prediction: “Probably Damaging”</td></tr>
<tr><td colspan="3"></td></tr>
</table>

PolyPhen 2: http://genetics.bwh.harvard.edu/pph2/

MutationTaster: https://www.mutationtaster.org/

gnomAD Browser: https://gnomad.broadinstitute.org/ . P112L and P112R are the only two (very rare) variants from the linker peptide observed in gnomAD. VUS: *variant of unknown significance*.

Appendix Table S3.

Location within NBS1/NBN	cBioPortal (TCGA dataset)	COSMIC
**FHA** *(aa1-109)*	P6S, A8T, G13*, E36A, R43Q, S72Y, M83Cfs*9, Q84H, R89Q, F99S, E109*, E109K.	W2C, P6S, A7S, A8T, G13*, R17T, G21C, E23K, E23V, Y24F, V25I, K29R, N30Tfs*5, I33F, E36A, E36D, N37T, Q39*, I41S, R43G, R43*, R43Q, A46V, F52L, L57Q, D61*, P64T, P64S, V65I, V65A, D70Y, S72Lfs*20, S72Y, G75C, E81K, K82E, M83CFS*9, Q84H, S88T, S88F, R89Q, S93A, S93L, G94V, D95N, G96S, G100A, G103V, G103Efs*6, K105E, E109*.
**Linker** *(aa110-114)*	--	E111* Reported in a single malignant melanoma. Sample: TCGA-EE-A29L-06
**BRCT$_1$** *(aa115-182)*	D121Y, T126A, T158I, I162V, R169H, L181M.	S118C, C119S, D121N, S123P, G124R, G124E, K125E, K125Rfs*34, T126A, T126S, A131D, Q134R, L135F, L135P, G136R, N142S, M152I, V153Kfs*17, V155M, V155F, T158I, I162V, L165H, I166T, R169C, R169H, P170L, I171V, K173*, T178I, L181M.

A survey of the cancer-specific NBS1/*NBN* variants with **FHA-Linker-BRCT$_1$** catalogued in the cBioPortal TCGA pan-cancer Atlas Studies dataset (https://www.cbioportal.org/) and in the Catalogue of Somatic Mutations in Cancer (COSMIC) portal (https://cancer.sanger.ac.uk/cosmic). R43Q is a recurrent variant listed 10x times in COSMIC. All other recurrent variants are underlined. Variants found in both cBioPortal and COSMIC are highlighted.

Appendix Table S4.

**Cell line**	**Fitness Score (*PPIA* KO)**	**BRCA2 status**	**Alteration**
**HCC38**	-12.5575	WT	-
**CAMA-1**	-12.3565	WT	-
**OCUB-M**	-11.747	WT	-
**HCC1954**	-10.942	WT	-
**CAL-51**	-10.942	Truncation	p.N986fs*5
**EVSA-T**	-8.815	WT	-
**MDA-MB-468**	-8.808	WT	-
**EFM-19**	-8.1255	CNA	***BRCA2*** CN Gain. Total CN=4 *(Ploidy: 3.019)*
**HCC1143**	-7.224	CNA	***BRCA2*** **CN Loss**. Total CN=2 *(Ploidy: 3.931)*
**BT-549**	-7.0075	CNA	***BRCA2*** CN Gain. Total CN=4 *(Ploidy: 3.073)*
**HCC70**	-6.246	CNA	***BRCA2*** **CN Loss**. Total CN=3 *(Ploidy: 4.494)*
**MDA-MB-361**	-6.2415	Missense VUS	p. N1657S<br>Polyphen2 score: 0.144 = *Benign*
**HCC1395**	-6.1745	Truncation<br>CNA	p.E1593*<br>***BRCA2*** CN Gain. Total CN=4 *(Ploidy: 3.069)*
**UACC-893**	-6.1745	CNA	***BRCA2*** **CN Loss**. Total CN=2 *(Ploidy: 3.112)*
**AU565**	-5.4785	WT	-
**DU-4475**	-5.3675	WT	-
**HCC1419**	-4.531	CNA	***BRCA2*** **CN Loss**. Total CN=1 *(Ploidy: 2.084)*
**COLO-824**	-4.39	Missense VUS	p.D1902N<br>PolyPhen2 score: 0.055 = *Benign*
**Hs-578-T**	-4.314	WT	-
**JIMT-1**	-4.17	WT	-
**MDA-MB-157**	-4.093	Missense VUS<br>CNA	p.L929S & p.N987I<br>Polyphen2 scores: 0.091 = *Benign* & 0.744 = *Possibly damaging*, respectfully.<br>***BRCA2*** **CN Loss**. Total CN=2 *(Ploidy: 2.85)*
**HCC202**	-3.831	CNA	***BRCA2*** **CN Loss**. Total CN=2 *(Ploidy: 3.038)*
**SUM-229PE**	-3.736	*No data*	-

**HMC-1-8**	-3.638	CNA	***BRCA2*** CN Gain. Total CN=5 *(Ploidy: 3.678)*
**SUM-52PE**	-2.581	*No data*	-
**MDA-MB-231**	-2.5535	WT	-
**MFM-223**	-2.21	CNA	***BRCA2*** **CN Loss**. Total CN=2 *(Ploidy: 2.669)*
**HCC1187**	-2.1255	WT	-
**HCC1806**	-1.79	Missense VUS<br><br>CNA	p.Y42C & p.L929S & p.N987I<br><br>PolyPhen2 scores: 0.09 = *Benign* & 0.091 = *Benign* & 0.744 = *Possibly damaging*, respectfully.<br><br>***BRCA2*** CN Gain. Total CN=4 *(Ploidy: 2.335)*
**SK-BR-3**	-1.596	*No data*	-
**HCC1428**	-1.5065	CNA	***BRCA2*** **CN Loss**. Total CN=2 *(Ploidy: 3.725)*
**SUM-159PT**	-1.2735	*No data*	-
**T47D**	-0.5505	CNA	***BRCA2*** **CN Loss**. Total CN=2 *(Ploidy: 2.77)*
**HCC1937**	-0.1345	WT	-
**ZR-75-1**	0.3205	WT	-
**CAL-120**	0.748	CNA	***BRCA2*** **CN Loss**. Total CN=3 *(Ploidy: 4.579)*
**SUM-149PT**	1.654	*No data*	-
**MDA-MB-453**	2.703	CNA	***BRCA2*** CN Gain. Total CN=3 *(Ploidy: 2.197)*
**MDA-MB-436**	3.0855	WT	-
**MDA-MB-415**	3.478	Missense VUS	p.T598A<br><br>PolyPhen2 score: 0.001 = *Benign*
**MCF7**	4.522	WT	-

PPIA/CYPA CRISPR knockout Fitness Scores from the DepMap *Project Score* (https://score.depmap.sanger.ac.uk/) for a range of breast carcinoma cell lines. CNA: *copy number aberration*. CN: *copy number*. VUS: *variant of unknown significance*. Ploidy was determined by WES: *whole exome sequencing*. Individual cell line *BRCA2* status was extracted from the *Cell Model Passports* (https://cellmodelpassports.sanger.ac.uk/). In this cell line set, the two lines with *BRCA2* truncating variants (i.e., CAL-51 and HCC1395) both have significantly reduced loss of fitness upon *PPIA* knockout. Except for p.N987I (found in MDA-MB-157 and HCC1806; both of whom show loss of fitness upon *PPIA* loss), all other *BRCA2* VUS are predicted (by PolyPhen2) to be benign. Of the lines showing reduced fitness upon *PPIA/*CYPA loss, 9/34 (26.5%) exhibit *BRCA2* CN loss compared to 1/7 (14.3%) lines where *PPIA/* CYPA knockout does not impact fitness.

Appendix Table S5.

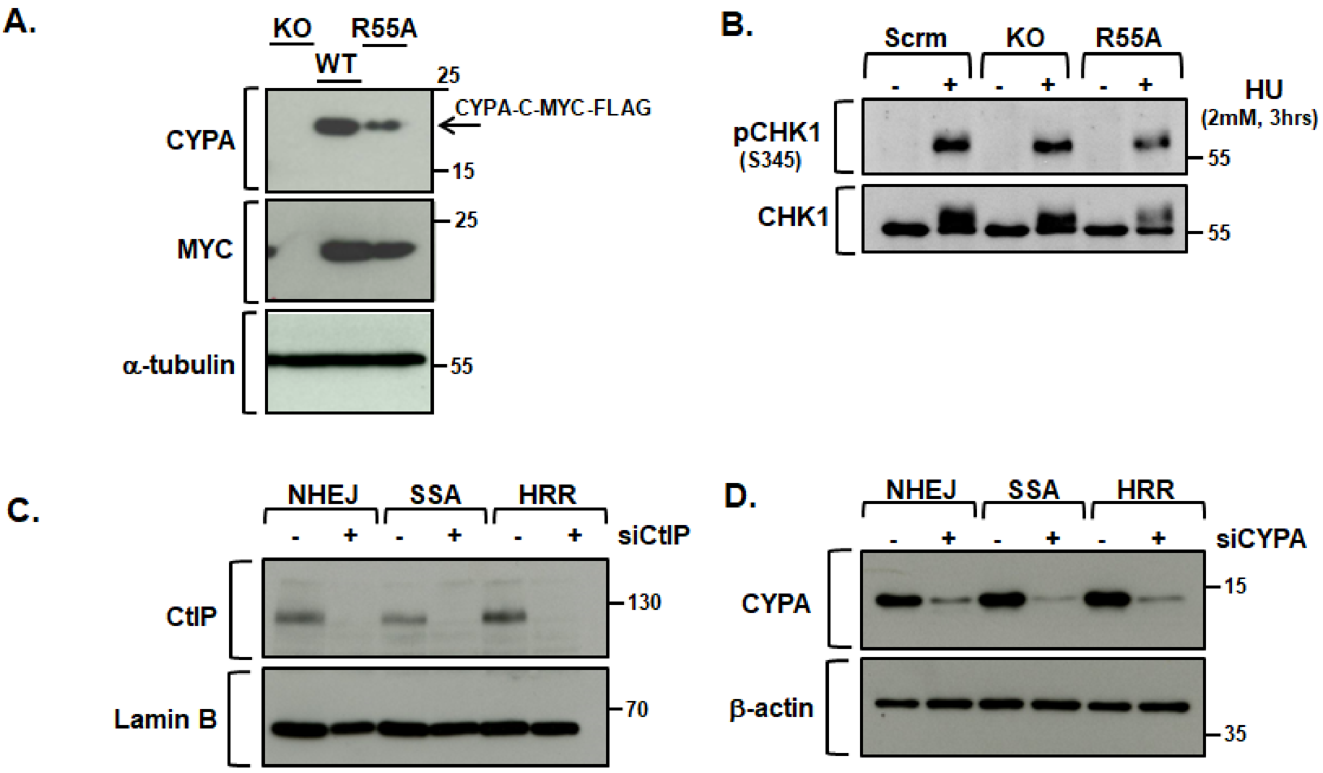

Appendix Figure S1.

A. USOS cells CRISPR engineered *PPIA*/CYPA-knockout (KO) and reconstituted with wild-type (WT) or isomerase-dead (p.R55A) CYPA (R55A). The upper panel shows western blotting for CYPA expression. The middle panel shows detection of the WT and R55A CYPA using the MYC tag. The lower panel confirms protein expression across all three backgrounds: KO, WT, R55A.

B. HU-induced (2mM, 3hrs) ATR-dependent CHK1 phosphorylation (pS345) is comparable in scrambled control (Scrm), CYPA-KO and CYPA-R55A lines.

C. Western blot analysis confirming reduction in CtIP expression in the DR-GFP DSB-R reporter cell lines following siCtIP.

D. Western blot analysis confirming reduction in CYPA expression in the DR-GFP DSB-R reporter cell lines following siCYPA.

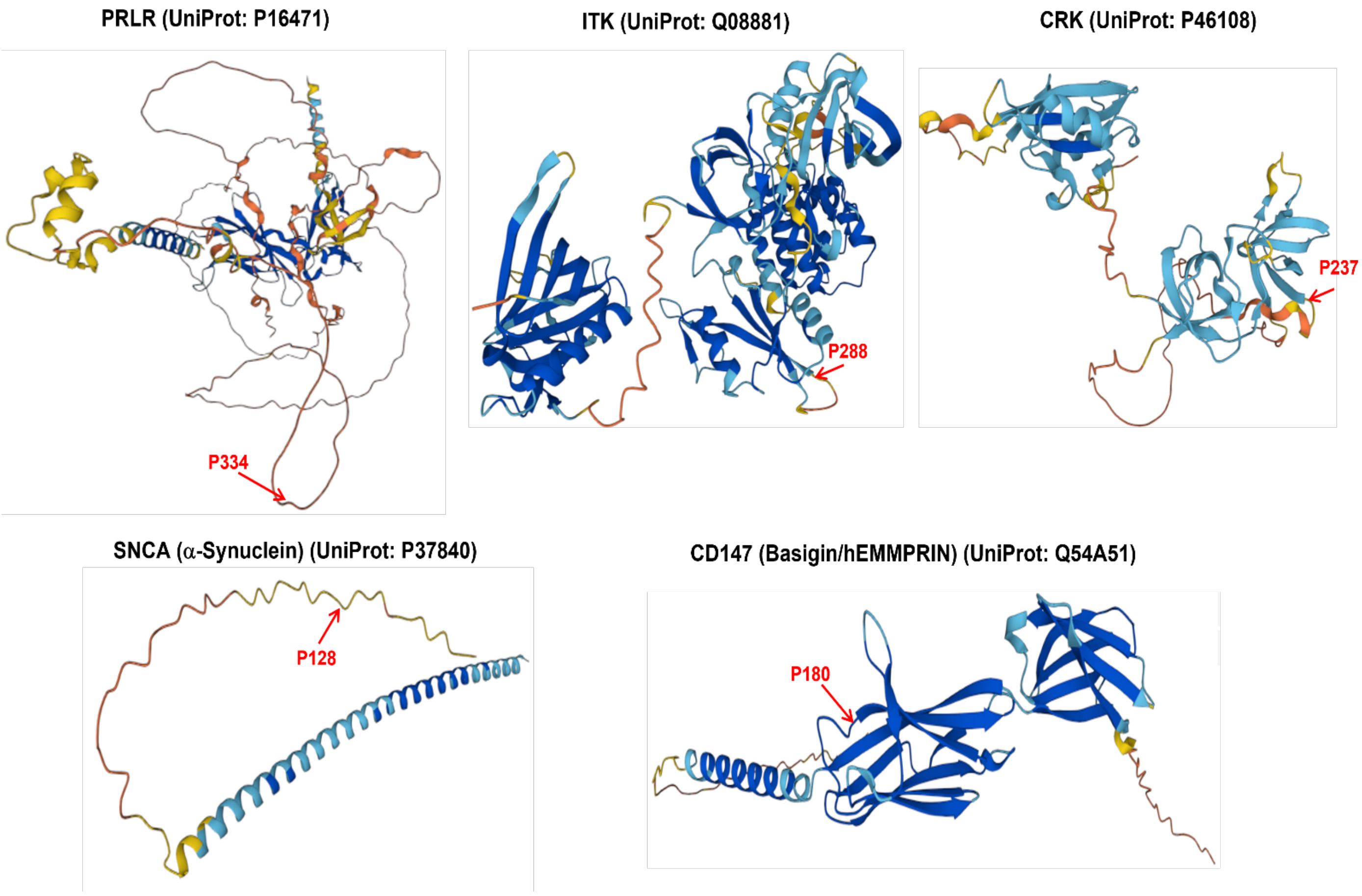

Appendix Figure S2.

AlphaFold-derived structures of verified CYPA interactors, indicating the relative positioning of their specific prolyl *cis-trans* isomerase proline target residues. These are typically located in regions of disorder (e.g., PRLR, SCNA) or in disordered regions between two areas of order (e.g., ITK, CRK, CD147).

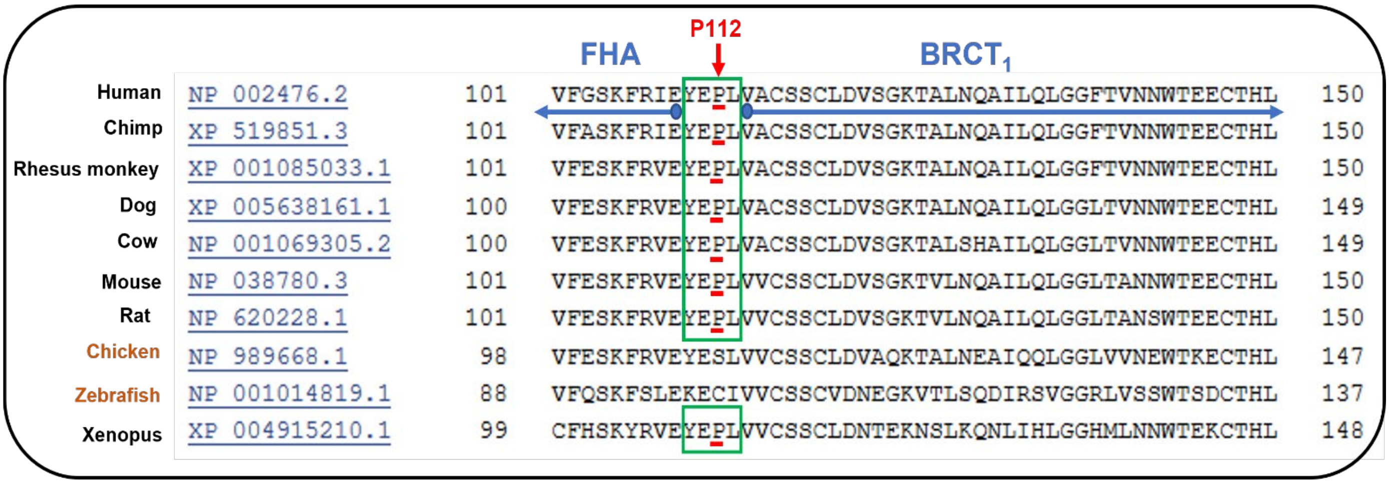

Appendix Figure S3.

MUSCLE alignment of part of NBS1 FHA and part of the adjoining BRCT1 including the linker region, showing the high degree of conservation of the linker sequence and its resident P112 (human) equivalent amongst a range of vertebrates. Of note, P112 is not conserved in *S.pombe* (not shown), from where much of the structural insight of FHA domains and MRN/MRX were originally derived.

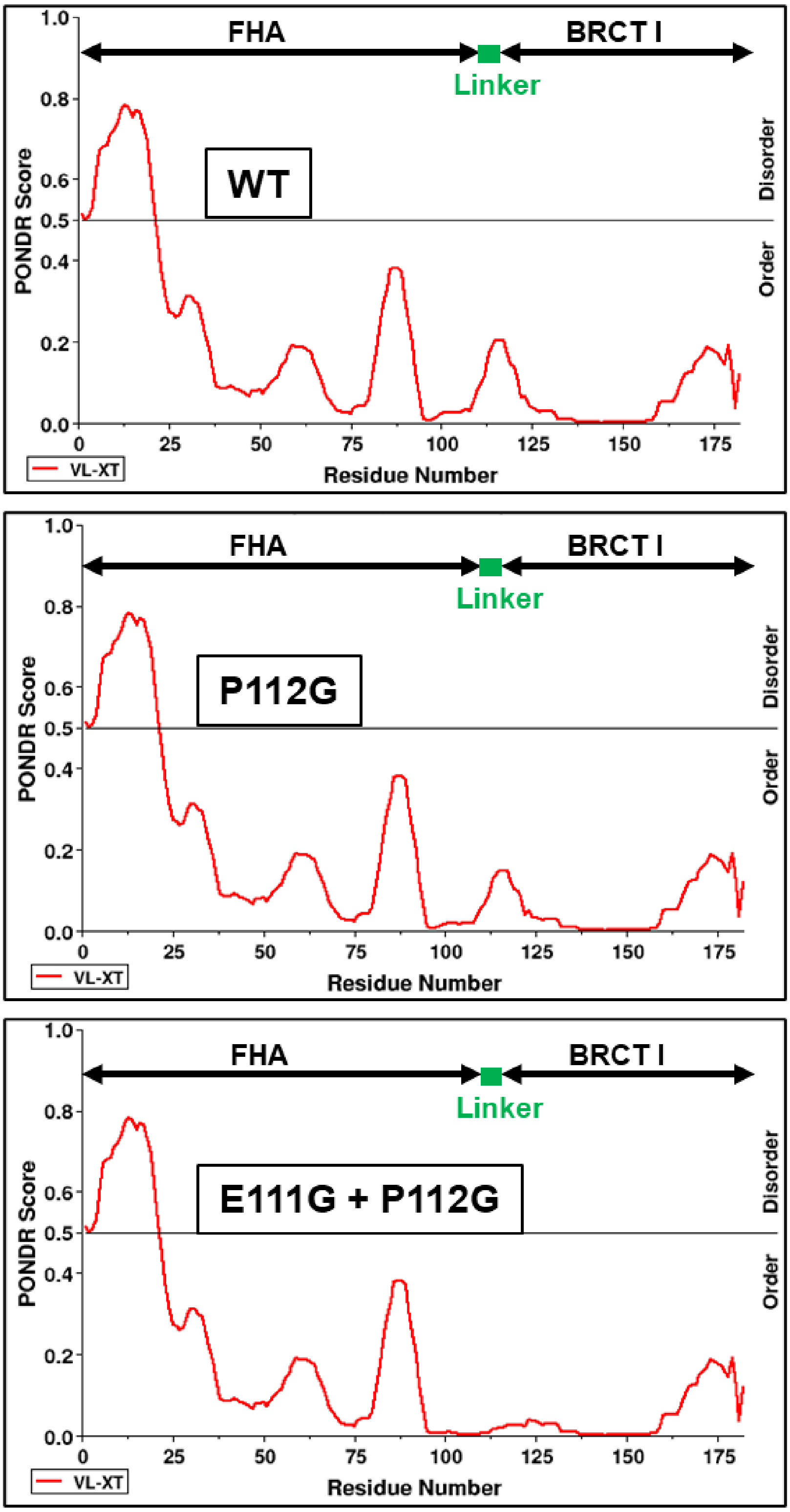

Appendix Figure S4.

PONDR (Predictor of Natural Disordered Regions) analysis profiles of various NBS1 peptides spanning aa1-182 and incorporating FHA-linker-BRCT$_1$. No tangible differences are observed between the profiles, indicating that ablation of the linker residues P112 or E111+P112 does not alter the order of this region.

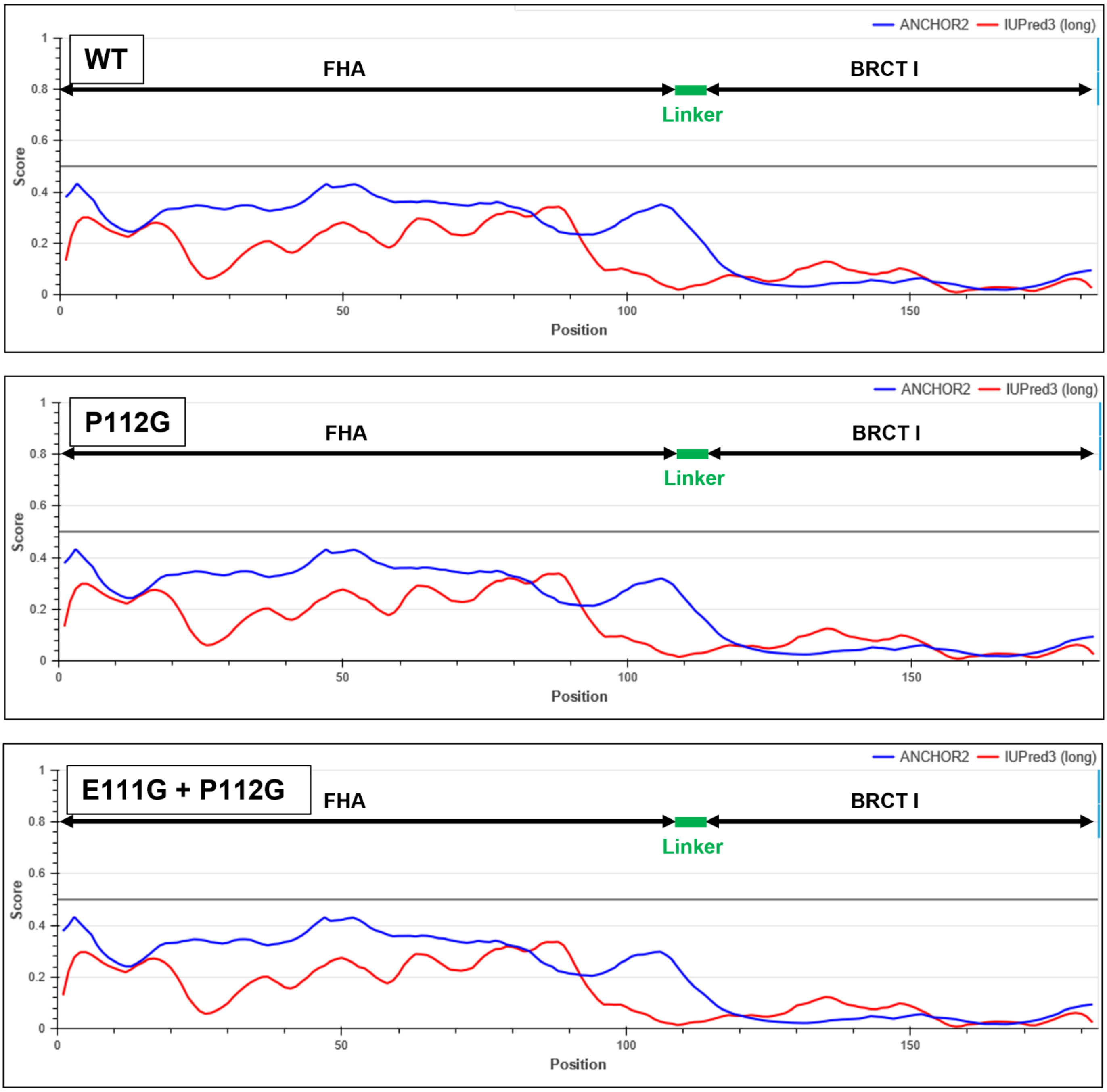

Appendix Figure S5.

IUPRED3-derived profiles (ANCHOR2 and IUPRED3 analysis) of various NBS1 peptides spanning aa1-182 and incorporating FHA-linker-BRCT$_1$. No tangible differences are observed between the profiles, indicating that ablation of the linker residues P112 or E111+P112 does not alter the order of this region.

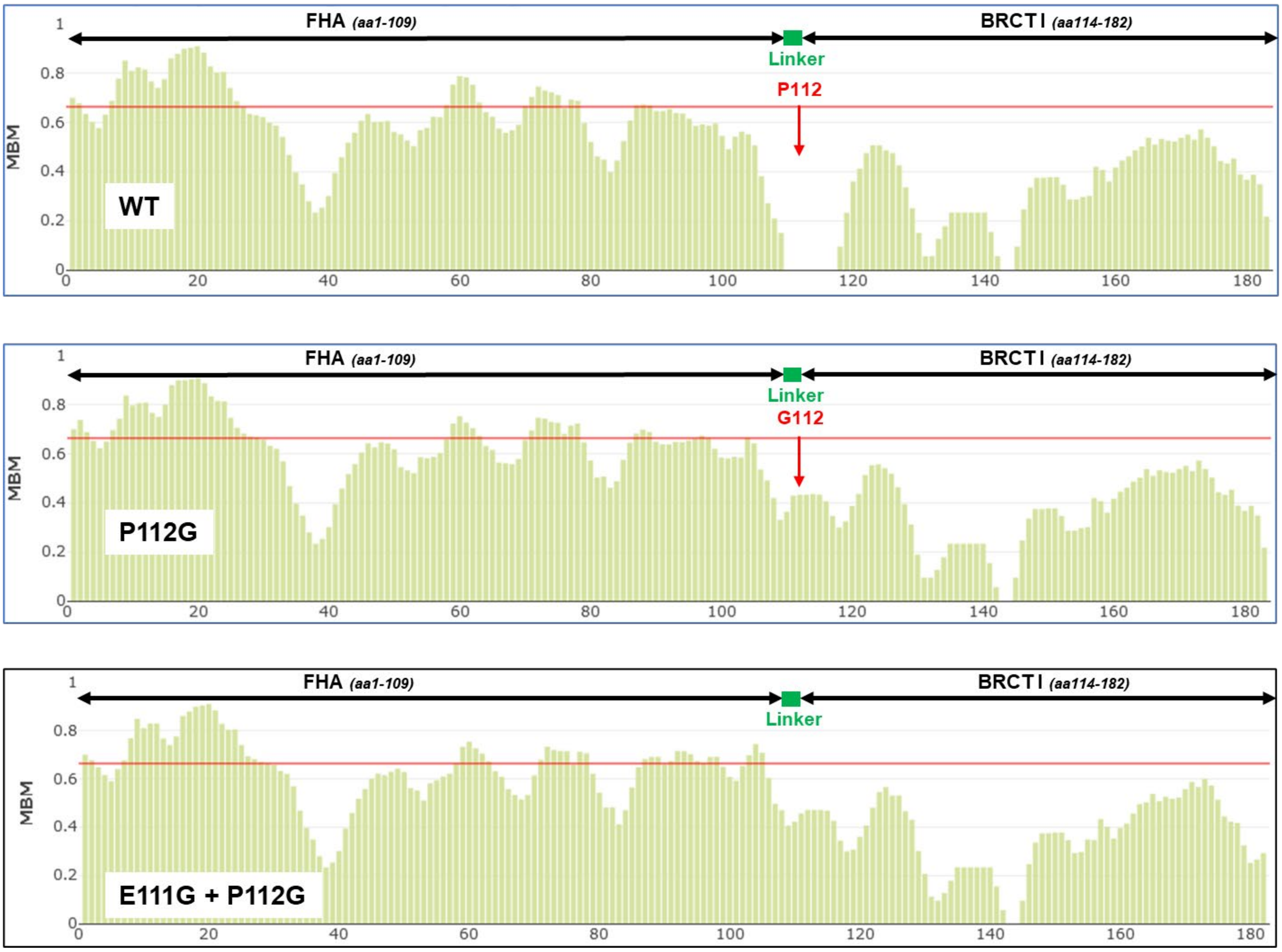

Appendix Figure S6.

FuzzPred analysis profiles of various NBS1 peptides spanning aa1-182 and incorporating FHA-linker-$BRCT_1$. Although slight differences in multiple binding modality (MBM) are evident around the linker region with P112G or E111G+P112G compared to the wild-type peptide, these local changes didn't attain significance (as indicated by residue signals being above the red line).

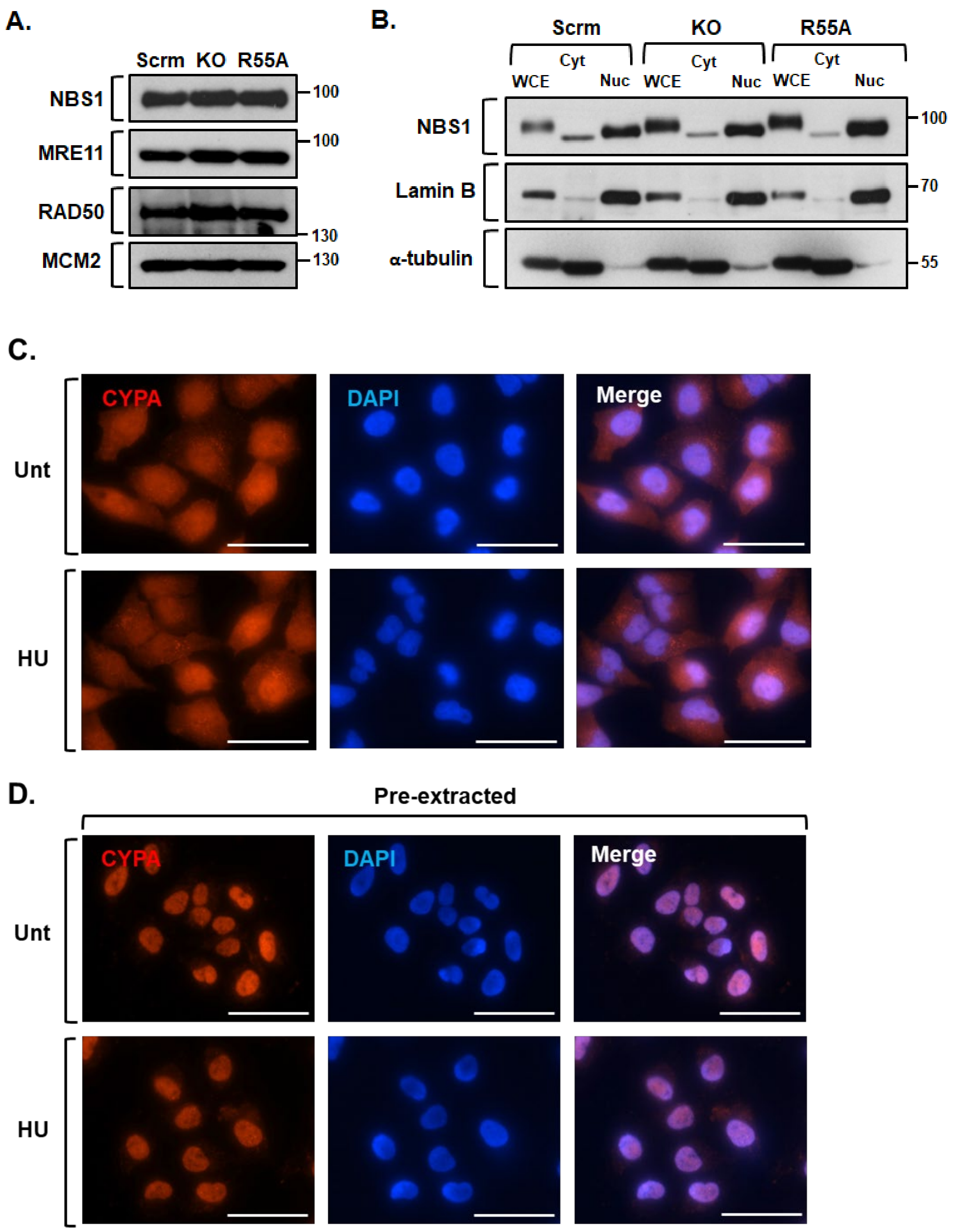

Appendix Figure S7.

A. Expression of NBS1, MRE11 and RAD50 is equivalent in scrambled control (Scrm), CYPA-KO and CYPA-R55A cell lines demonstrating that loss and/or inhibition of CYPA does not have any stochastic impacts upon MRN expression and/or stability.

B. The subcellular distribution of NBS1 is comparable in scrambled control (Scrm), CYPA-KO and CYPA-R55A cell lines. WCE: *whole cell extract*. Cyt: *cytosplasmic extract*. Nuc: *nuclear extract*. Lamin B was used as a nuclear marker whilst expression of α-tubulin was used as a cytoplasmic marker to attest to the quality of the fractionation.

C. Indirect immunofluorescence (IF) of CYPA in untreated (Unt) and HU-treated (HU: 2mM, 3hrs) U2OS cells. Nuclei were counterstained with DAPI. CYPA staining appears as nuclear and cytoplasmic under both conditions. Scale bar: 50μm.

D. IF of CYPA in untreated (Unt) and HU-treated (HU: 2mM, 3hrs) U2OS cells following pre-extraction with Triton X100 prior to fixation. CYPA staining appears nuclear in Unt and HU treated cells. CYPA does not form discernible foci under these conditions. Nuclei were counterstained with DAPI. Scale bar: 50μm.

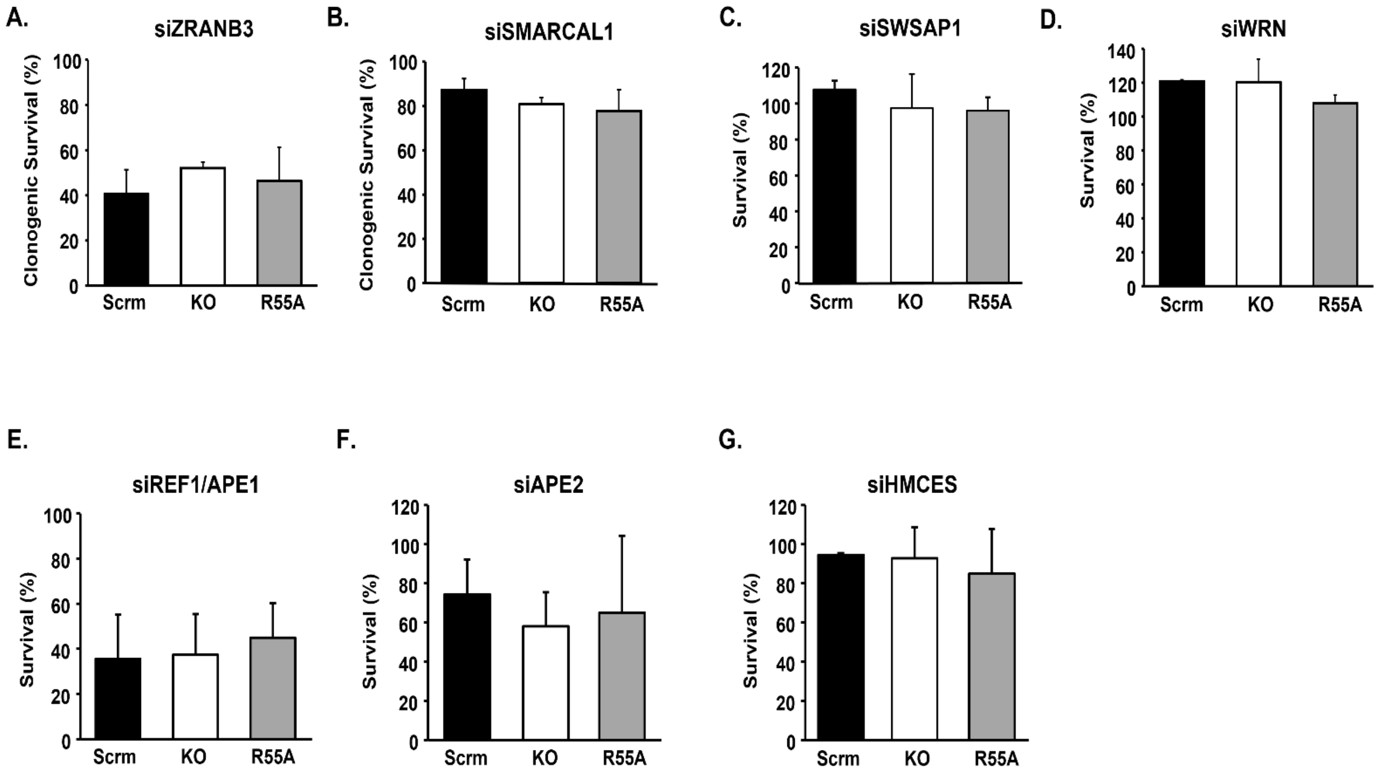

Appendix Figure S8.

Survival analysis following siRNA in the U2OS isogenic panel of cell lines engineered to be Scrm; scrambled control, KO; *PPIA*/CYPA knockout and R55A; KO reconstituted with CYPA-p.R55A. We observed no significant impact in clonogenic survival or viability upon siRNA of any of these targets when comparing the Scrm cell line to either KO or R55A isogenic U2OS lines *(error bars represent the mean ± s.d. of at least three independent experiments)*.

A. Clonogenic survival following siZRANB3.

B. Clonogenic survival following siSMARCAL1.

C. Crystal violet survival analysis following siSWSAP1.

D. Crystal violet survival analysis following siWRN.

E. Crystal violet survival analysis following siREF1/APE1.

F. Crystal violet survival analysis following siAPE2.

G. Crystal violet survival analysis following siHMCES.

Appendix Materials & Methods.

Appendix Table S6: ***Cell lines.***

Cell line	Source	Culture media
**HEK293 (CRL-1573)**	American Type Culture Collection (ATCC)	DMEM supplemented with 10% foetal bovine serum (FBS), L-glutamate (2mM) and antibiotics, penicillin-streptomycin (1%).
**U2OS (HTB-96) and derivatives**	ATCC	DMEM…*as above*.
**AA8**	Genome Damage & Stability (GDSC) Cell Bank, Uni of Sussex.	DMEM…*as above*
**irs1SF**	GDSC Cell Bank, Uni of Sussex.	DMEM…*as above*
**CHO-K1**	GDSC Cell Bank, Uni of Sussex.	DMEM…*as above*
**xrs-6**	GDSC Cell Bank, Uni of Sussex.	DMEM…*as above*
**H1299-shBRCA (Dox-inducible)**	Prof. Madalena Tarsounas, Dept of Oncology, Uni of Oxford, UK (as described in Tacconi EMC *et al* EMBO Mol Med 2017)	DMEM…*as above*
**NBS-ILB1**	Dr. Malgorzata Z Zdzienicka, MGC-Dept of Radiation Genetics & Chemical Mutagenesis, Leiden University-LUMC, Netherlands (originally described in Kraakman-van der Zwer M *et al* Mut Res 1999)	DMEM…*as above*
**SH-SY5Y**	European Collection of Authenticated Cell Cultures (ECACC).	DMEM : F12 (1:1) supplemented with 10% FBS L-glutamate (1%) and antibiotics, penicillin-streptomycin (1%).
**SK-N-SH**	ECACC	DMEM supplemented with 10% FBS, L-glutamate (2mM) and antibiotics, penicillin-streptomycin (1%).
**KELLY**	ECACC	RPMI 1640 supplemented with 10% FBS, L-glutamate (2mM) and antibiotics, penicillin-streptomycin (1%).
**IMR-32**	ECACC	EMEM (EBSS) supplemented with 10% FBS, 2mM Glutamine and 1% Non-Essential Amino Acids (NEAA)
**SK-N-DZ**	ECACC	DMEM supplemented with 10% FBS, 2mM Glutamine and 1% Non-Essential Amino Acids (NEAA)
**CHP-143**	ECACC	RPMI 1640 supplemented with 10% FBS, L-glutamate (2mM) and antibiotics, penicillin-streptomycin (1%).
**AG87 (AG09387)**	Coriell Cell Repositories	RPMI 1640 supplemented with 15% FBS, L-glutamate (2mM) and antibiotics, penicillin-streptomycin (1%).
**KG1**	ATCC	RPMI 1640 supplemented with 10% FBS, L-glutamate (2mM) and

		antibiotics, penicillin-streptomycin (1%).
**MM.1S (CRL-2974)**	ATCC	RPMI 1640 supplemented with 10% FBS, L-glutamate (2mM) and antibiotics, penicillin-streptomycin (1%).
**RPMI-8226 (CRL-155)**	ATCC	RPMI 1640 supplemented with 10% FBS, L-glutamate (2mM) and antibiotics, penicillin-streptomycin (1%).
**KG1 (CCL-246)**	ATCC	RPMI 1640 supplemented with 10% FBS, L-glutamate (2mM) and antibiotics, penicillin-streptomycin (1%).
**Nalm6 (CRL-3273)**	ATCC	RPMI 1640 supplemented with 10% FBS, L-glutamate (2mM) and antibiotics, penicillin-streptomycin (1%).
**K562 (CCL-243)**	ATCC	Iscove's Modified Dulbecco's Medium supplemented with 10% FBS, L-glutamate (2mM) and antibiotics, penicillin-streptomycin (1%).
**TK6 (CRL-1815)**	ATCC	RPMI 1640 supplemented with 10% FBS, L-glutamate (2mM) and antibiotics, penicillin-streptomycin (1%).

All lines have been STR verified and confirmed to be mycoplasma free at time of use.

Appendix Table S7: ***Antibodies.***

Antibody target	Source	Cat Number
**CYPA** ***(for IP)***	Abcam	ab58144
**CYPA** ***(for IF)***	Genetex	GTX104698
**MYC**	Santa Cruz Biotech	Sc-40
**α-tubulin**	Merck-SiGMA	T5168
**Lamin B**	Santa Cruz Biotech	(C20):sc-6216
**PCNA**	Santa Cruz Biotech	(PC10):sc-56
**53BP1**	Bethyl	A300-272A
**CHAMP1**	Bethyl	A304-216A
**ILF2**	Santa Cruz Biotech	(H-4):sc-365283
**ILF3**	Bethyl	A303-119A-T
**pS4/pS8-RPA2**	Bethyl	A300-245A
**RPA2**	Calbiochem	NA18
**RAD51**	Santa Cruz Biotech	(H-92):sc-8349
**NBS1** ***(for IF)***	Santa Cruz Biotech<br>Cell Signalling Biotech	(B-5):sc-515069<br>#14956
**NBS1**	Santa Cruz Biotech	(A-2):sc374168
**MRE11** ***(for IF)***	Novus	NB100-142
**MRE11**	Santa Cruz Biotech	(18):sc-135992
**RAD50**	Cell Signalling Biotech	#3427
**FLAG**	Merck-SIGMA	F3165
**HIS**	Clontech	631212
**MDC1**	Novus	NB100-395
**CtIP**	Bethyl	A300-488A
**RAD52**	Santa Cruz Biotech	(F-7):sc-365341
**β-actin**	Cell Signalling Biotech	#49675
**XRCC3**	Santa Cruz Biotech	(10F1/6):sc-53471
**RAD51C**	Santa Cruz Biotech	(2H11):sc-56214
**MYCN**	Santa Cruz Biotech	(B8.4.B):sc53993
**p85-PARP**	Promega	G7241
**PARP**	BioRad	mcA1522G
**Anti-Rabbit IgG-Cy3** ***(for IF)***	Merck-SIGMA	C2306
**Anti-Mouse IgG-FITC** ***(for IF)***	Merck-SIGMA	F0257

Appendix Table S8: *siRNA.*

Gene target	Source	Cat number
PPIA	Horizon Discovery *(ON-TARGETplus SMARTpool)*	L-004979-04-0005
RAD51C	Horizon Discovery *(ON-TARGETplus SMARTpool)*	L-010534-00-0005
RAD52	Horizon Discovery *(ON-TARGETplus SMARTpool)*	L-011760-00-0005
CtIP/RBBP8	Horizon Discovery *(ON-TARGETplus SMARTpool)*	L-011376-00-0005
LIG4 (h)	Santa Cruz Biotech *(siRNA Target Pool)*	sc-37394
XRCC3 (h)	Santa Cruz Biotech *(siRNA Target Pool)*	sc-37403
ZRANB3 (h)	Santa Cruz Biotech *(siRNA Target Pool)*	sc-63042
SMARCAL1 (h)	Santa Cruz Biotech *(siRNA Target Pool)*	sc-94423
SWSAP1 (h)	Santa Cruz Biotech *(siRNA Target Pool)*	sc-97263
WRN (h)	Santa Cruz Biotech *(siRNA Target Pool)*	sc-36843
REF1/APE1 (h)	Santa Cruz Biotech *(siRNA Target Pool)*	sc-29470
APE2 (h)	Santa Cruz Biotech *(siRNA Target Pool)*	sc-61974
HMCES (h)	Santa Cruz Biotech *(siRNA Target Pool)*	sc-78041

Appendix Table S9: *Vectors and Site Directed Mutagenesis (SDM).*

**Mammalian Expression**	**Source**	**Vector backbone**
Cyclophilin A (*PPIA:* NM_021130)	Origene Cat# RC203307	pCMV6-Entry (PS100001) Kan^{R}, Neo^{R}, C-ter MYC-FLAG
p95 NBS1 (*NBN*: NM_002485)	Origene RC214682	pCMV6-Entry (PS100001) Kan^{R}, Neo^{R}, C-ter MYC-FLAG
**Bacterial Expression**	**Source**	**Vector backbone**
Cyclophilin A (*PPIA:* NM_021130)	pET-15b from Novogene Cat# 69661-3	Amp^{R}. 2x Strep II tags on the C-terminus.
NBS1-FHA and BRCT	pET-15b from Novogene Cat# 69661-3.	Amp^{R}. NBS1- FHA domain and BRCT1 cloned into pET-15b (aa 1-187) + 8xHIS on C-terminus.
**SDM Gene target**	**Forward oligo**	**Reverse oligo**
Mammalian expression: PPIA (R55A)	GTTCCTGCTTTCACgcAATTATTCCAGGG	CCCTGGAATAATTgcGTGAAAGCAGGAAC
Mammalian expression: NBN (NBS1) P112G	AGAGTATGAGggtTTGGTTGCATGC	ATTCTGAATTTACTTCCAAAC
Mammalian expression: NBN (NBS1) P64G	AGATGAAATCggtGTATTGACATTAAAAGATAATTCTAAG	GTTTGACTCAGGTTGGTTAC
Bacterial expression: NBN (NBS1) P112G	TGAGTACGAAgggCTCGTTGCTTGTTC	ATGCGAAACTTACTGCCG
