# Supplementary figures and images for: A novel role for the peptidyl-prolyl cis-trans isomerase Cyclophilin A in DNA-repair following replication fork stalling via the MRE11-RAD50-NBS1 complex
Source: EMBO Rep. 2024 Jun 28;25(8):3432–55. doi: 10.1038/s44319-024-00184-9 (PMC11315929; doi:10.1038/s44319-024-00184-9)

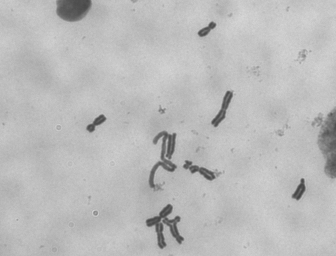

Supplement: Supplementary file 7 — Source data Fig. 1 [file 44319_2024_184_MOESM7_ESM.zip › Figure 1. Source Data/Fig 1A/Breaks & Fusions INSERT. Micr.image.tif]

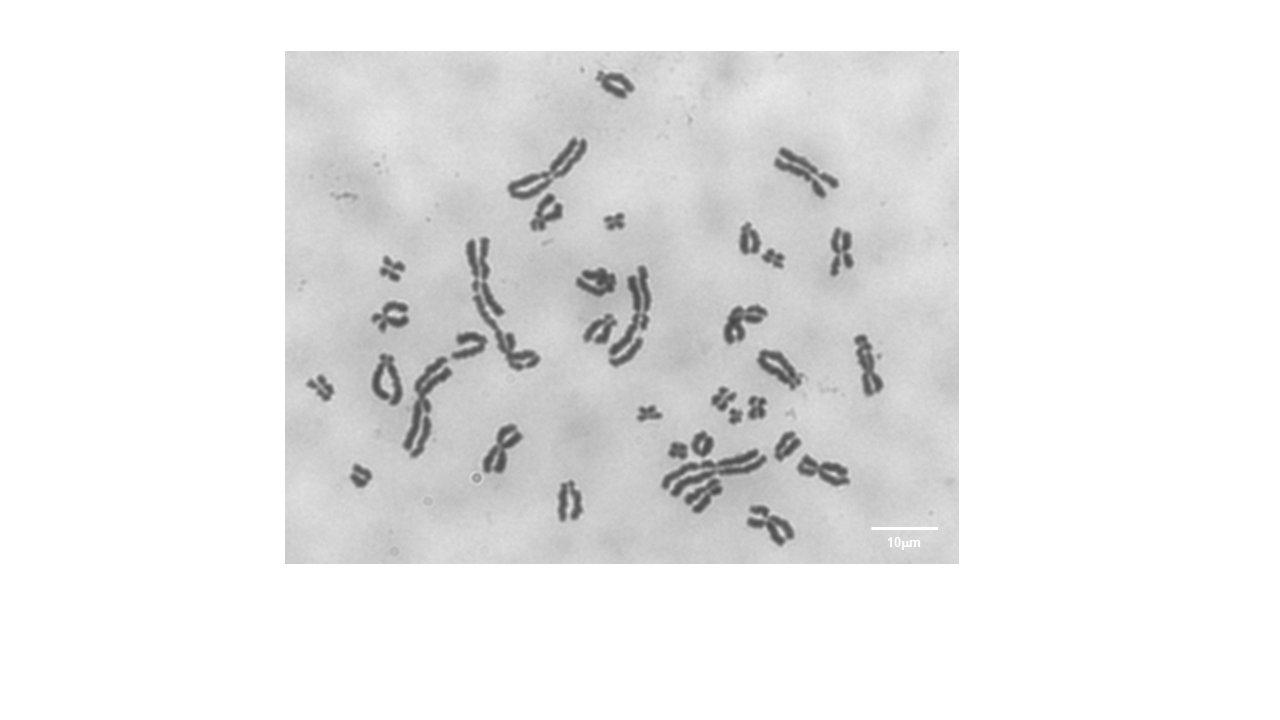

Supplement: Supplementary file 7 — Source data Fig. 1 [file 44319_2024_184_MOESM7_ESM.zip › Figure 1. Source Data/Fig 1A/Breaks & Fusions. Micr.image.tif]

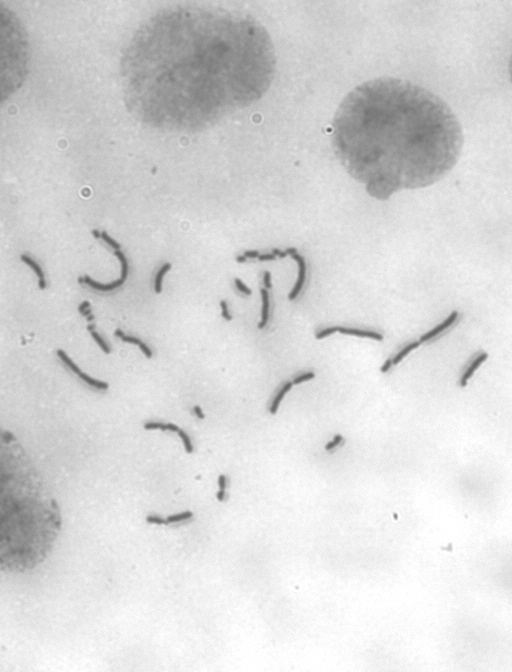

Supplement: Supplementary file 7 — Source data Fig. 1 [file 44319_2024_184_MOESM7_ESM.zip › Figure 1. Source Data/Fig 1A/SCEs. Micr.image.tif]

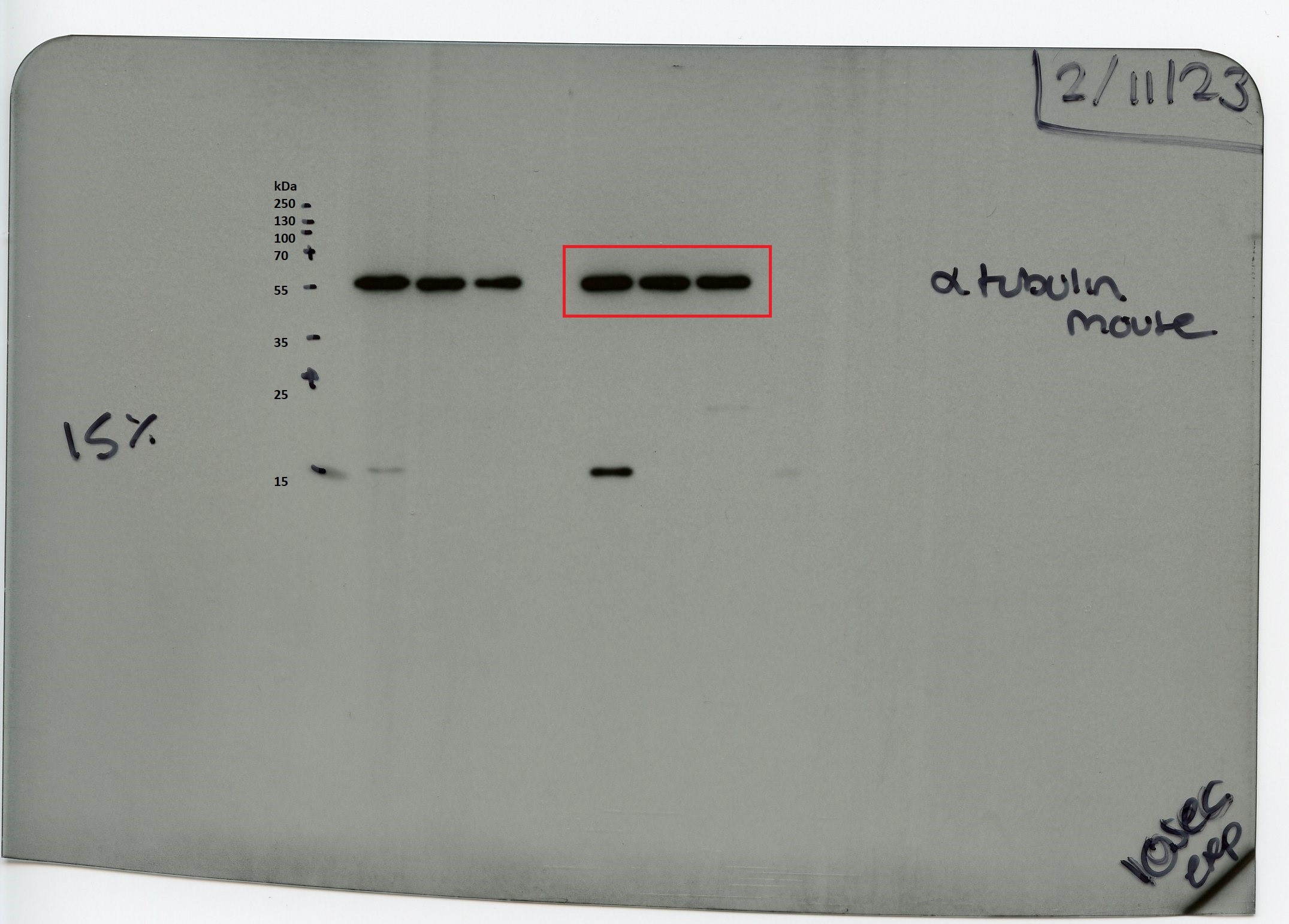

Supplement: Supplementary file 7 — Source data Fig. 1 [file 44319_2024_184_MOESM7_ESM.zip › Figure 1. Source Data/Fig 1C/Image data. Blot. alpha tubulin.jpg]

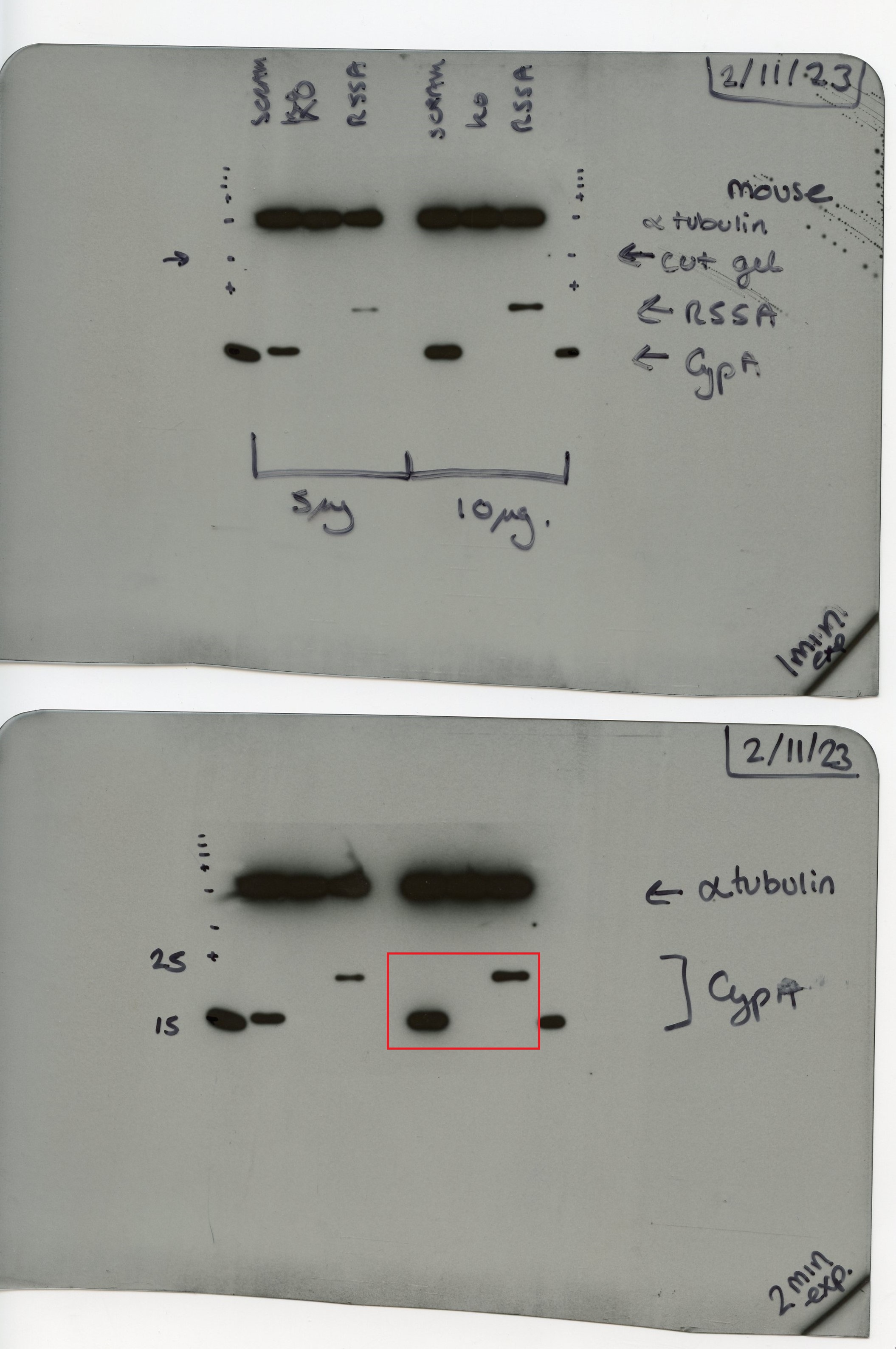

Supplement: Supplementary file 7 — Source data Fig. 1 [file 44319_2024_184_MOESM7_ESM.zip › Figure 1. Source Data/Fig 1C/Image data. Blot. CYPA.jpg]

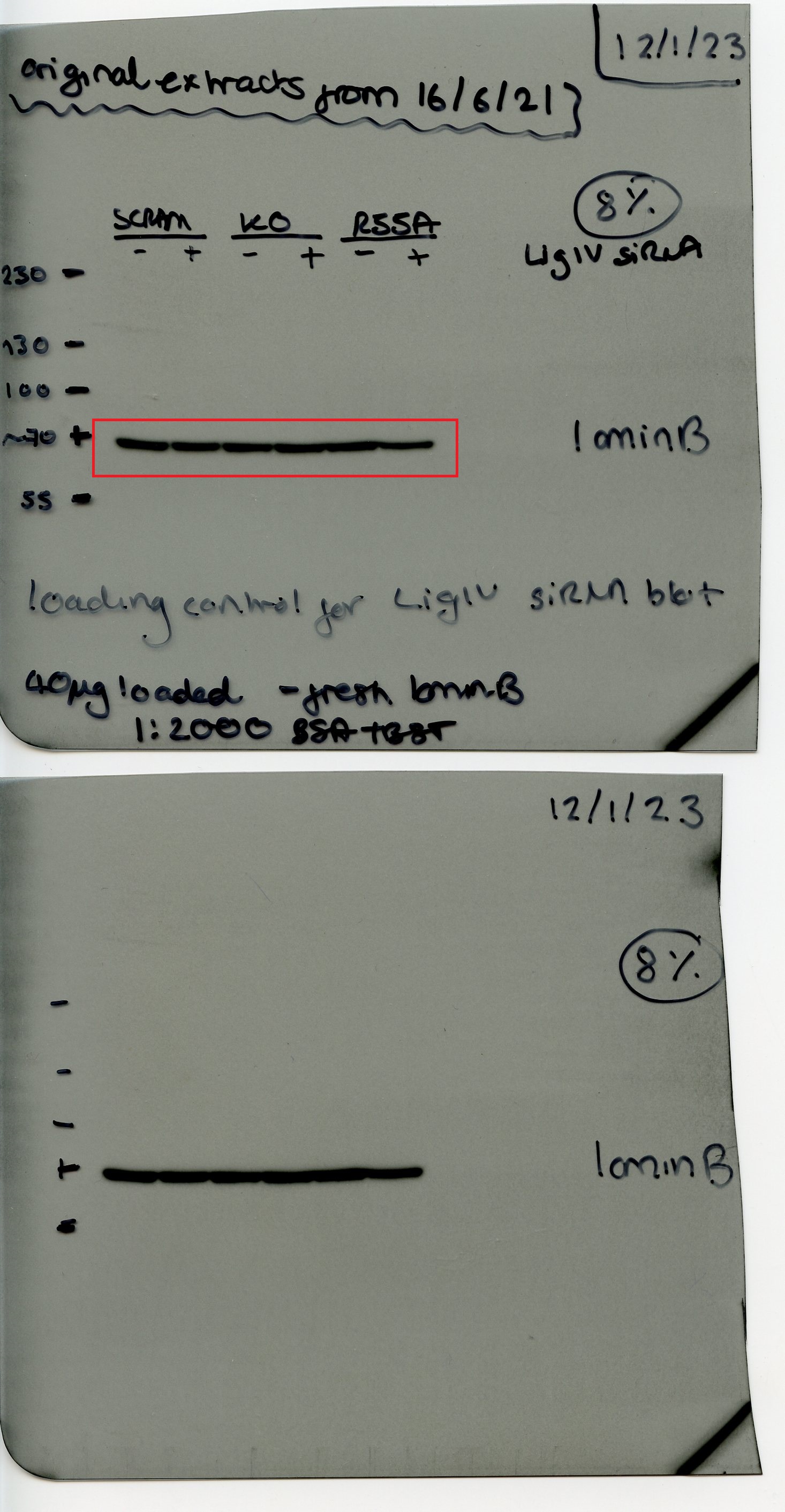

Supplement: Supplementary file 7 — Source data Fig. 1 [file 44319_2024_184_MOESM7_ESM.zip › Figure 1. Source Data/Fig 1D/Image data. Blot. Lamin B.jpg]

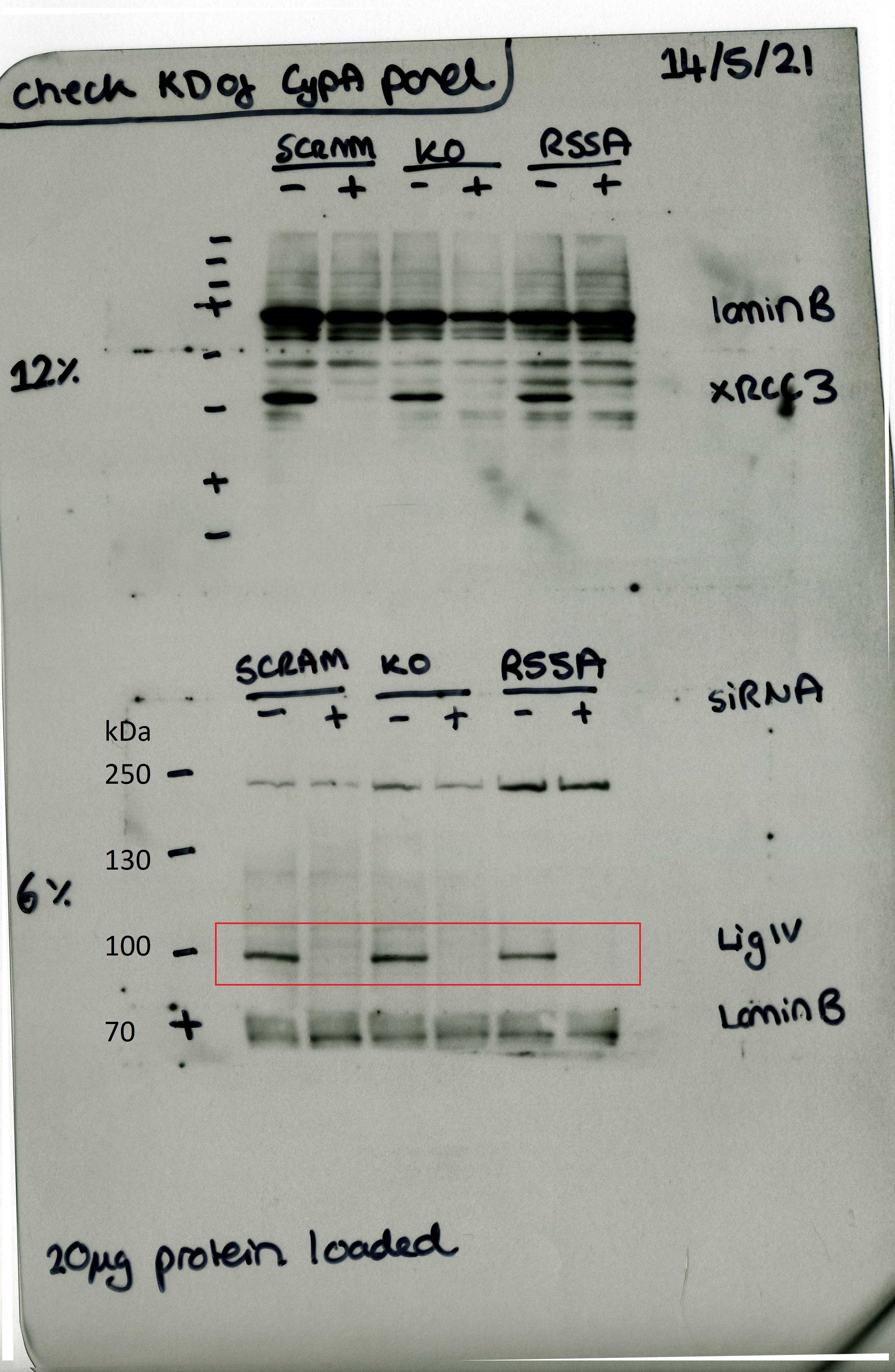

Supplement: Supplementary file 7 — Source data Fig. 1 [file 44319_2024_184_MOESM7_ESM.zip › Figure 1. Source Data/Fig 1D/Image data. Blot. LIG4.jpg]

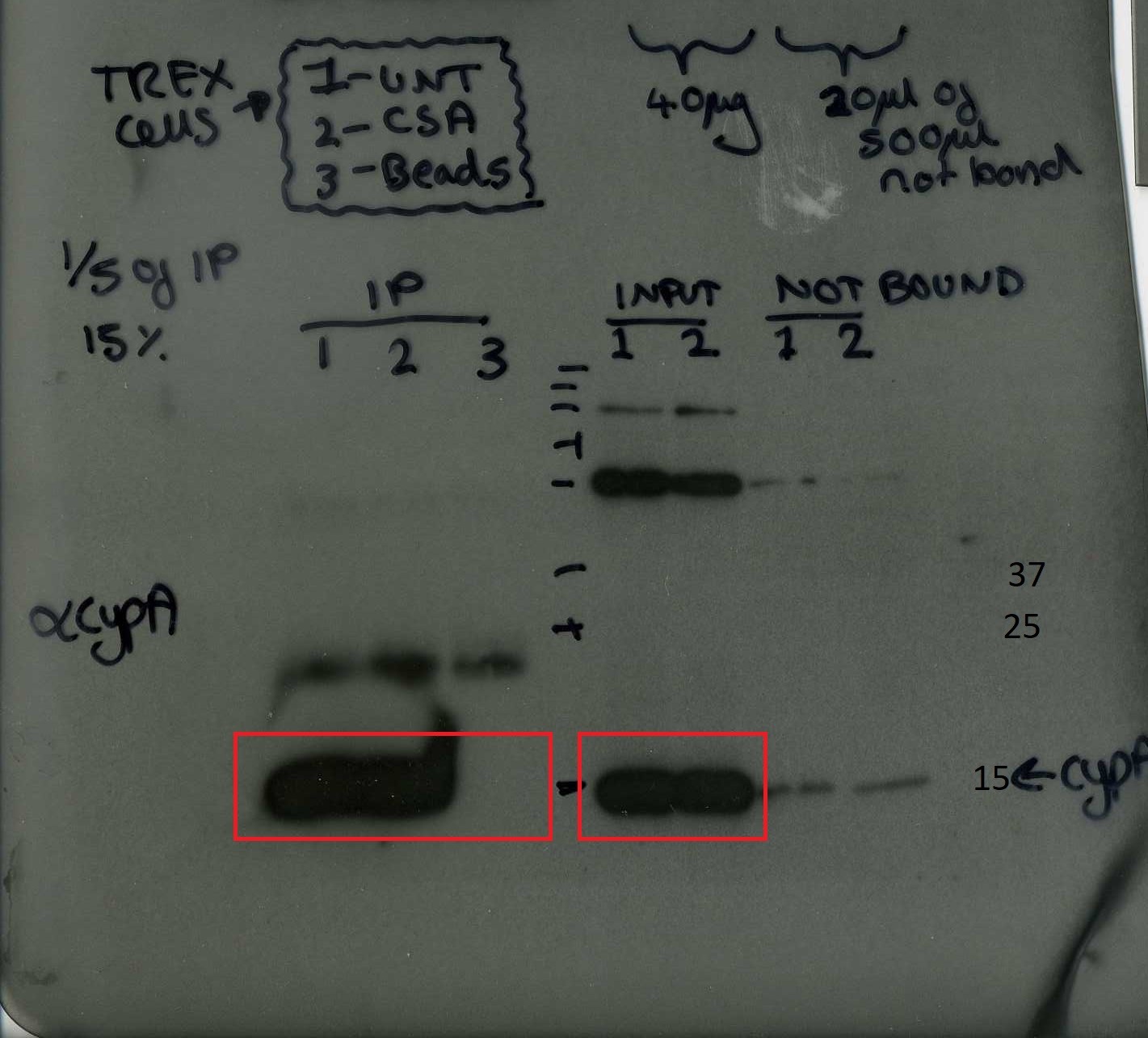

Supplement: Supplementary file 10 — Source data Fig. 5 [file 44319_2024_184_MOESM10_ESM.zip › Figure 5. Source Data/Fig 5B/Image data. Blot. CYPA coIP.jpg]

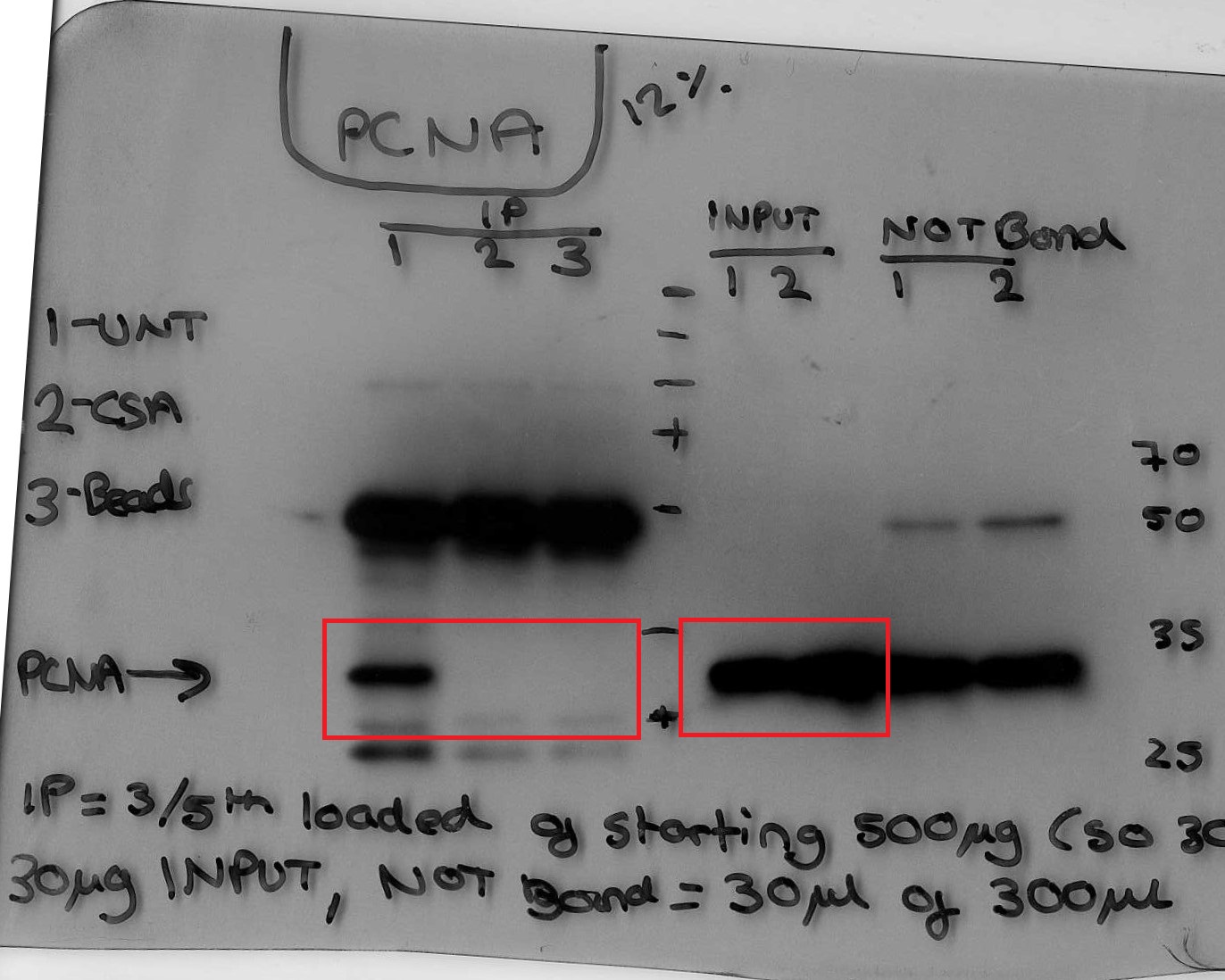

Supplement: Supplementary file 10 — Source data Fig. 5 [file 44319_2024_184_MOESM10_ESM.zip › Figure 5. Source Data/Fig 5B/Image data. Blot. PCNA coIP.jpg]

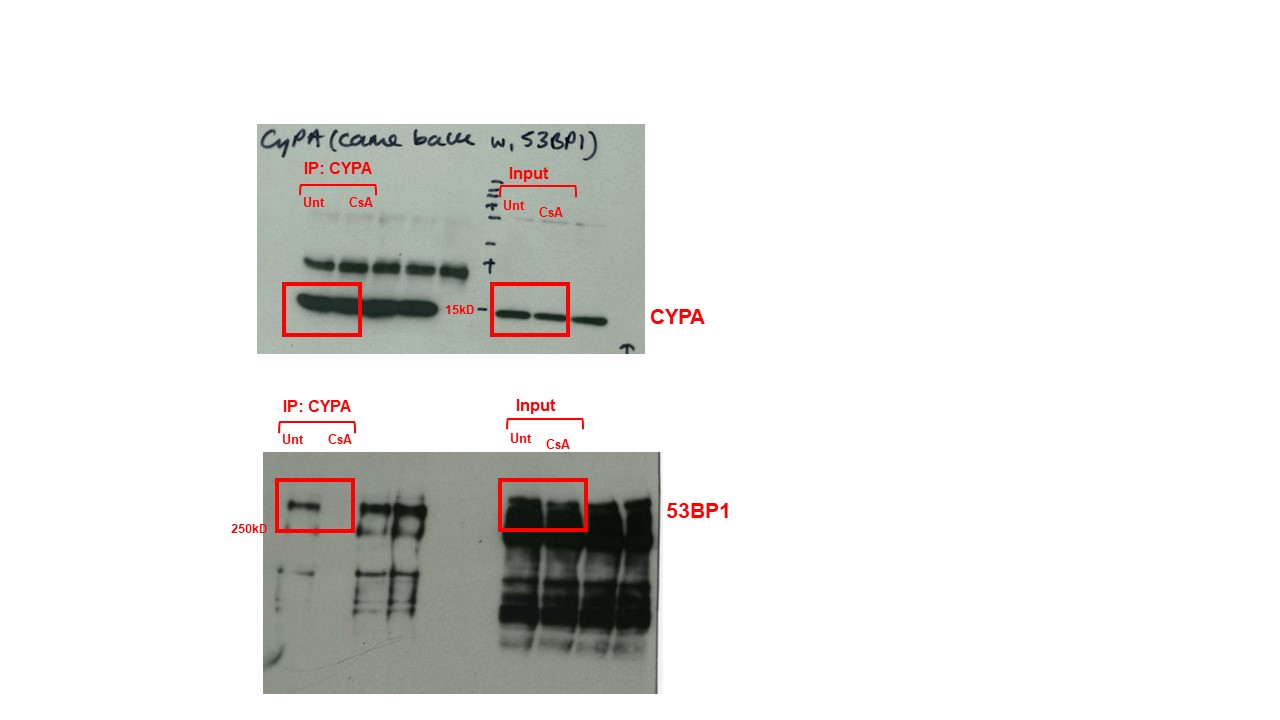

Supplement: Supplementary file 10 — Source data Fig. 5 [file 44319_2024_184_MOESM10_ESM.zip › Figure 5. Source Data/Fig 5C/Image data Blot. CYPA and 53BP1 coIP.jpg]

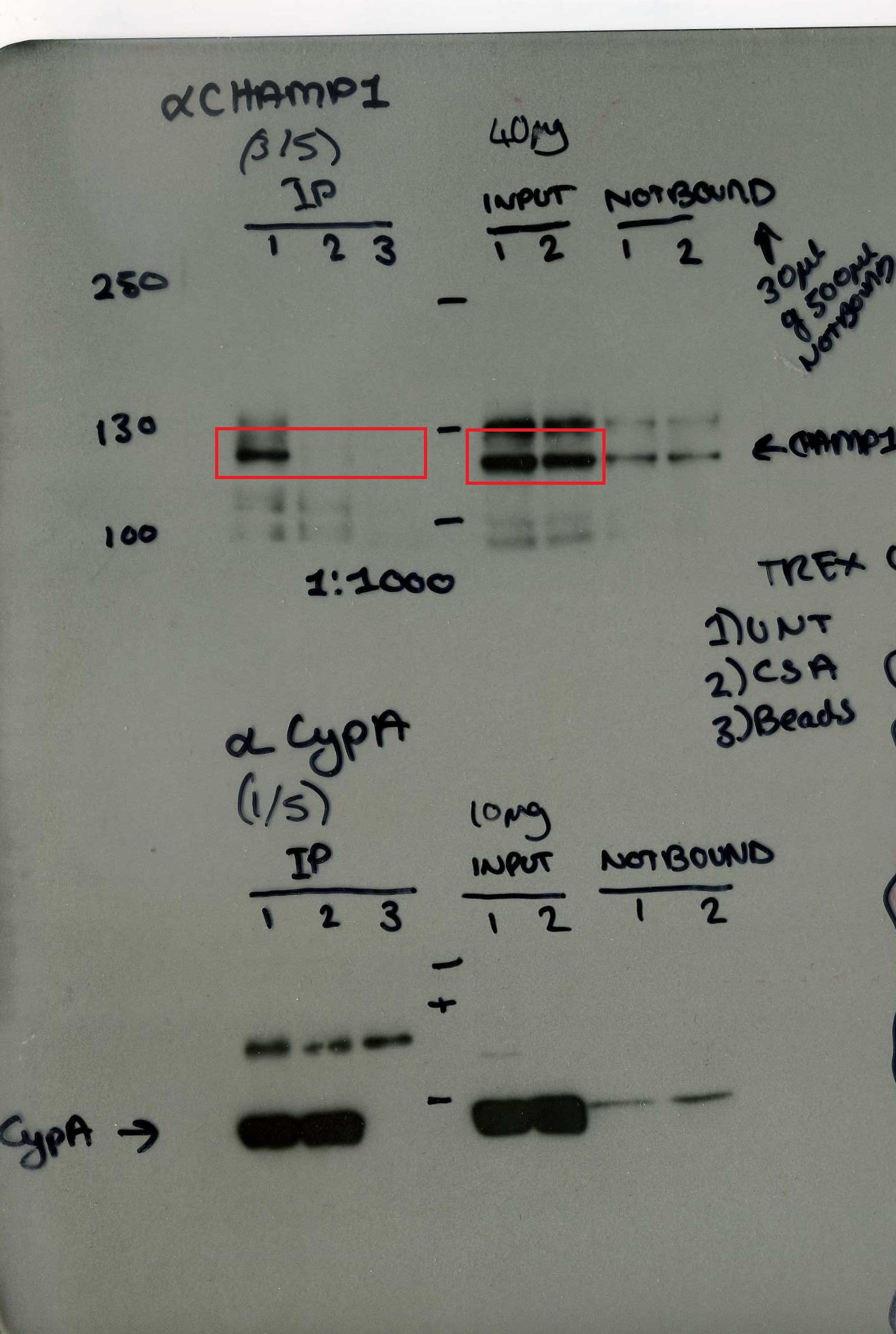

Supplement: Supplementary file 10 — Source data Fig. 5 [file 44319_2024_184_MOESM10_ESM.zip › Figure 5. Source Data/Fig 5D/Image data. Blot. CHAMP1 and CYPA coIP.jpg]

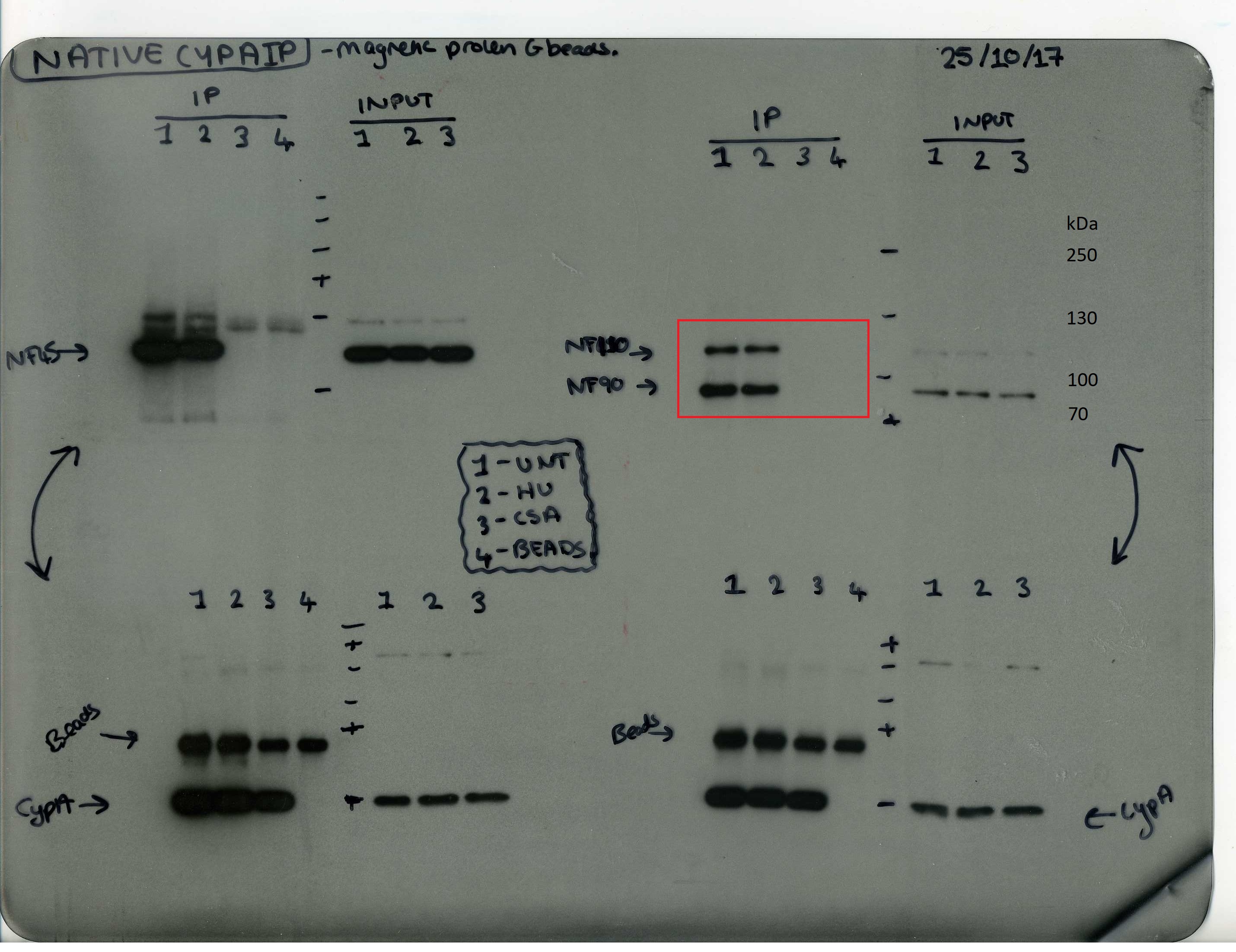

Supplement: Supplementary file 10 — Source data Fig. 5 [file 44319_2024_184_MOESM10_ESM.zip › Figure 5. Source Data/Fig 5E/Image data. Blot. ILF2_3 coIP CYPA no2.jpg]

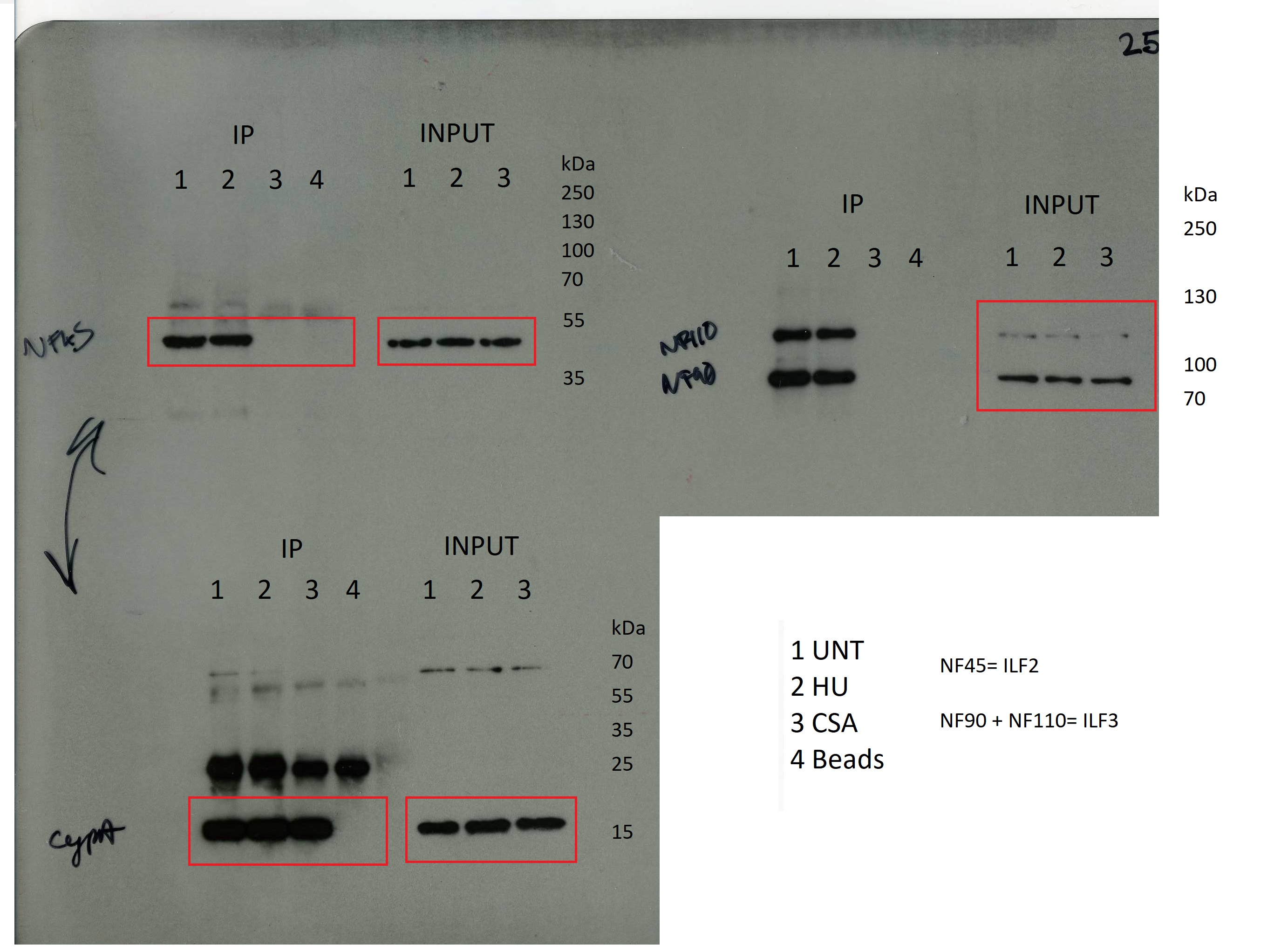

Supplement: Supplementary file 10 — Source data Fig. 5 [file 44319_2024_184_MOESM10_ESM.zip › Figure 5. Source Data/Fig 5E/Image data. Blot. ILF2_3 coIP CYPA.jpg]

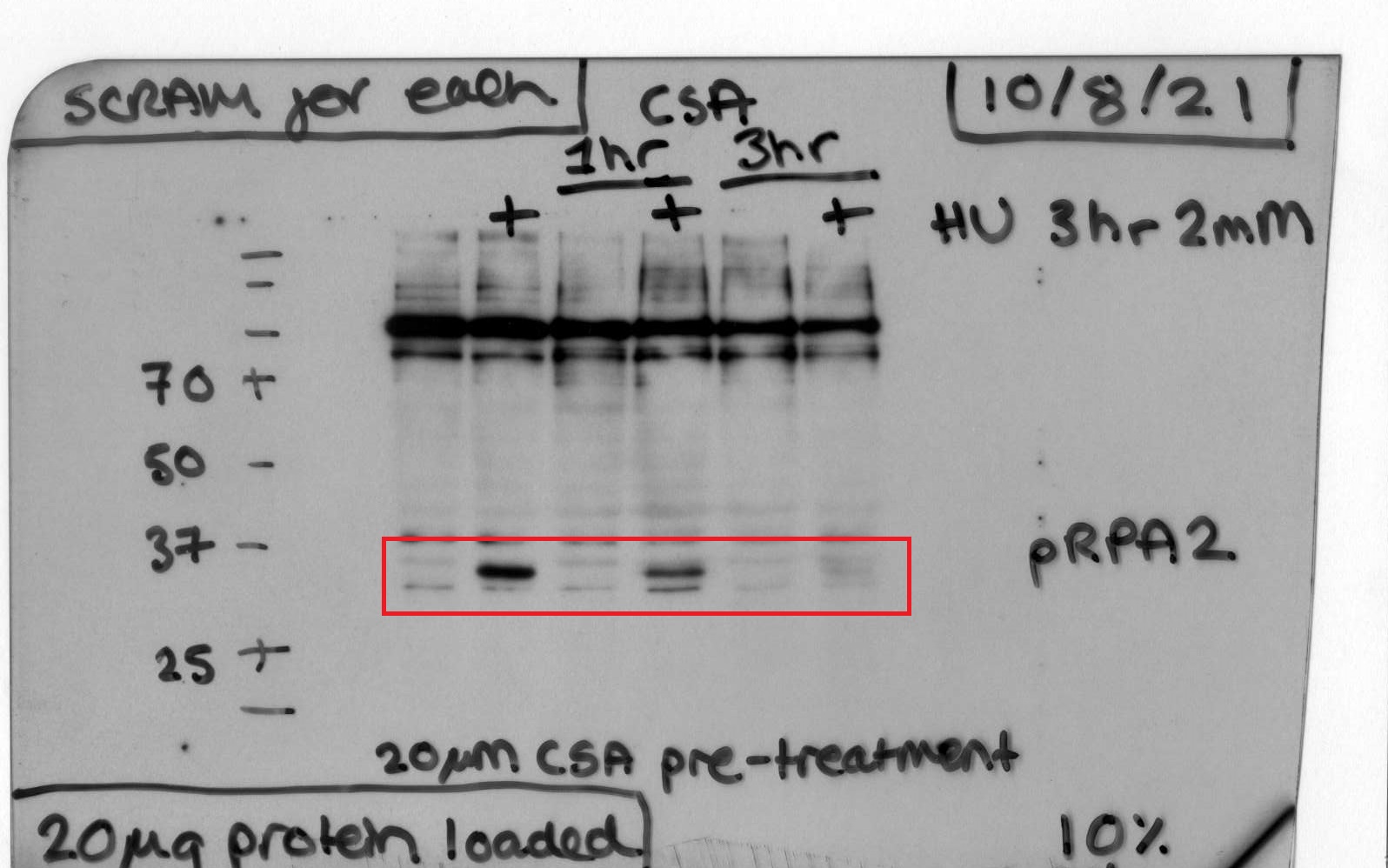

Supplement: Supplementary file 11 — Source data Fig. 6 [file 44319_2024_184_MOESM11_ESM.zip › Figure 6. Source Data/Fig 6A/Image data. Blot. pRPA2.jpg]

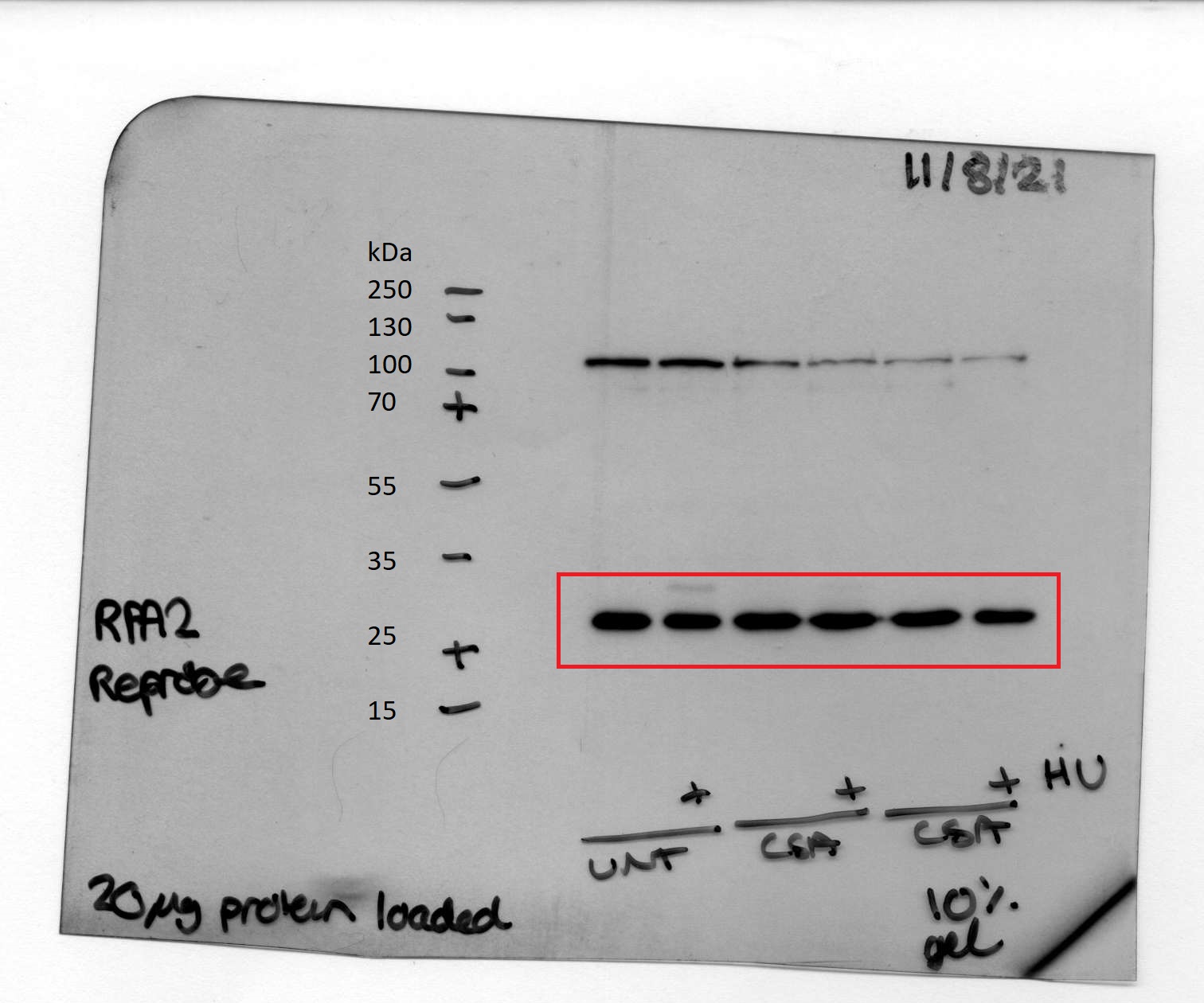

Supplement: Supplementary file 11 — Source data Fig. 6 [file 44319_2024_184_MOESM11_ESM.zip › Figure 6. Source Data/Fig 6A/Image data. Blot. RPA2.jpg]

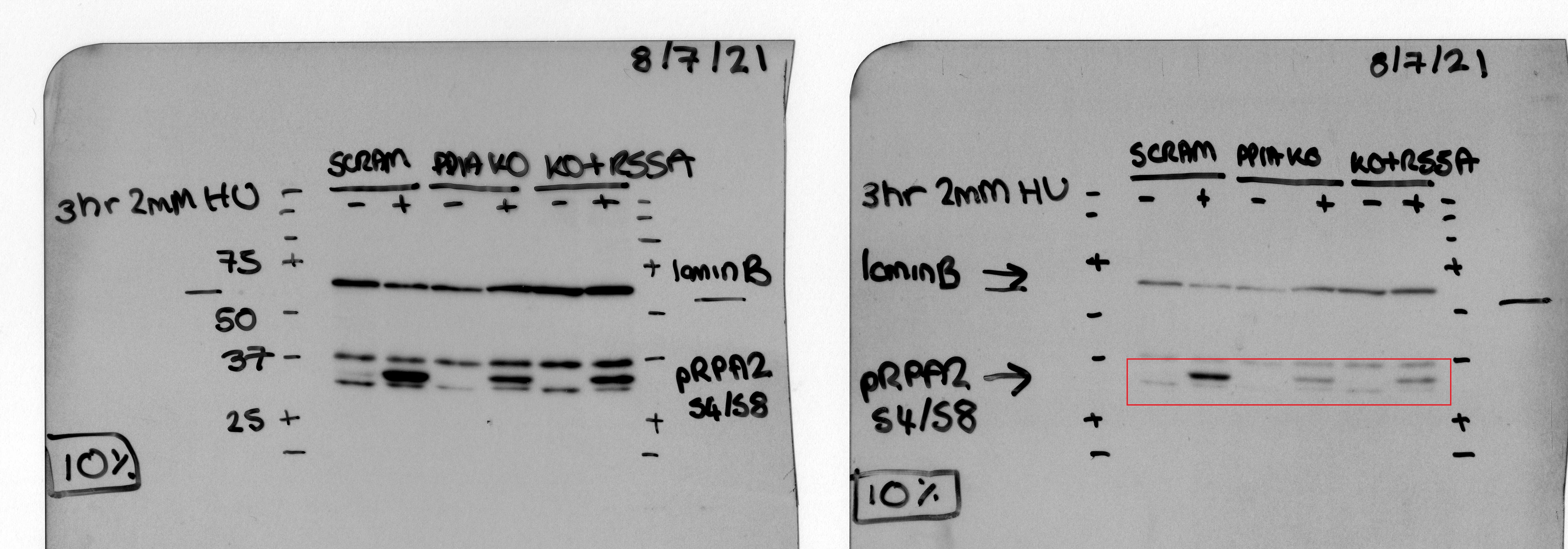

Supplement: Supplementary file 11 — Source data Fig. 6 [file 44319_2024_184_MOESM11_ESM.zip › Figure 6. Source Data/Fig 6B/Image data. Blot. pRPA2.jpg]

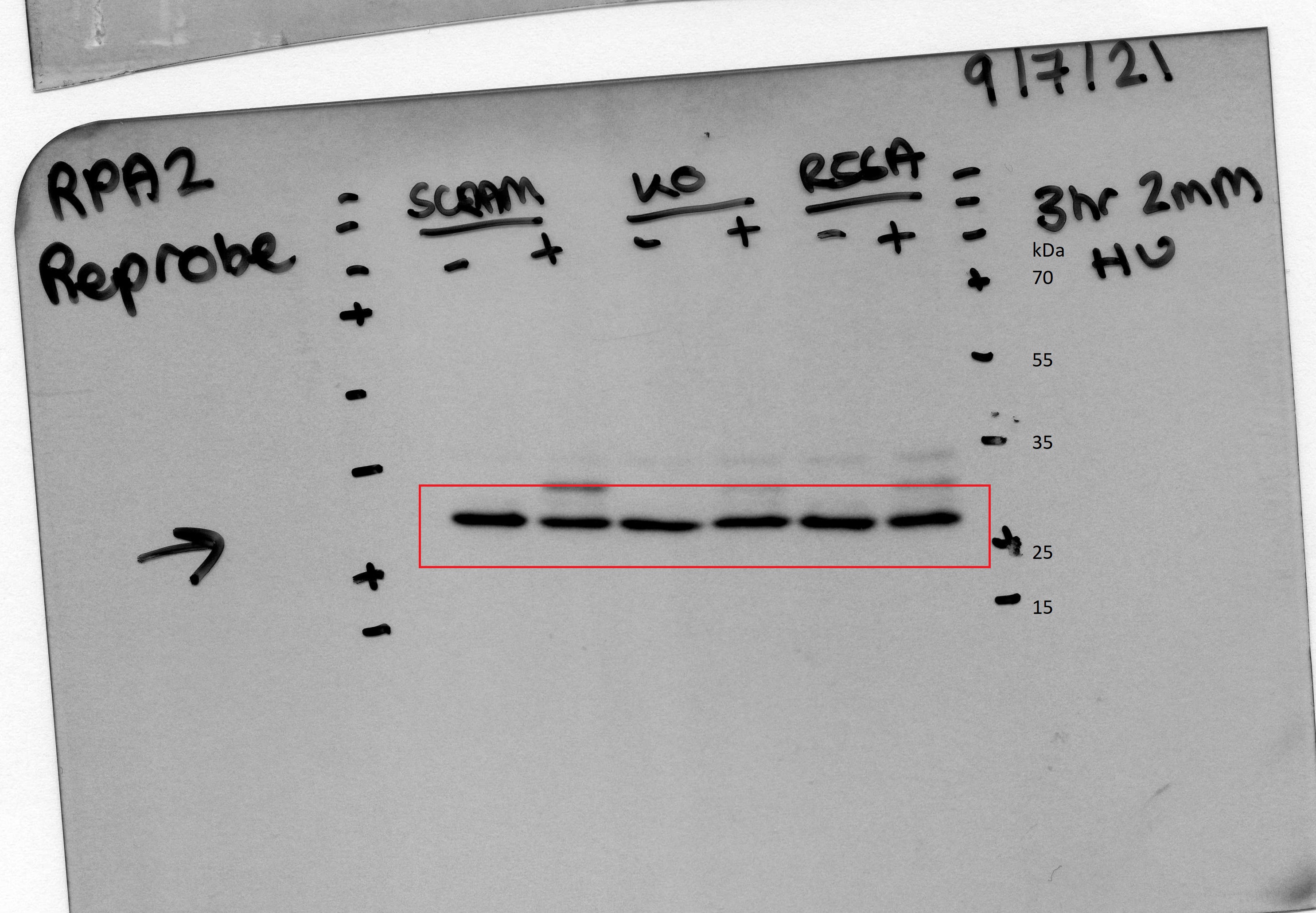

Supplement: Supplementary file 11 — Source data Fig. 6 [file 44319_2024_184_MOESM11_ESM.zip › Figure 6. Source Data/Fig 6B/Image data. Blot. RPA2.jpg]

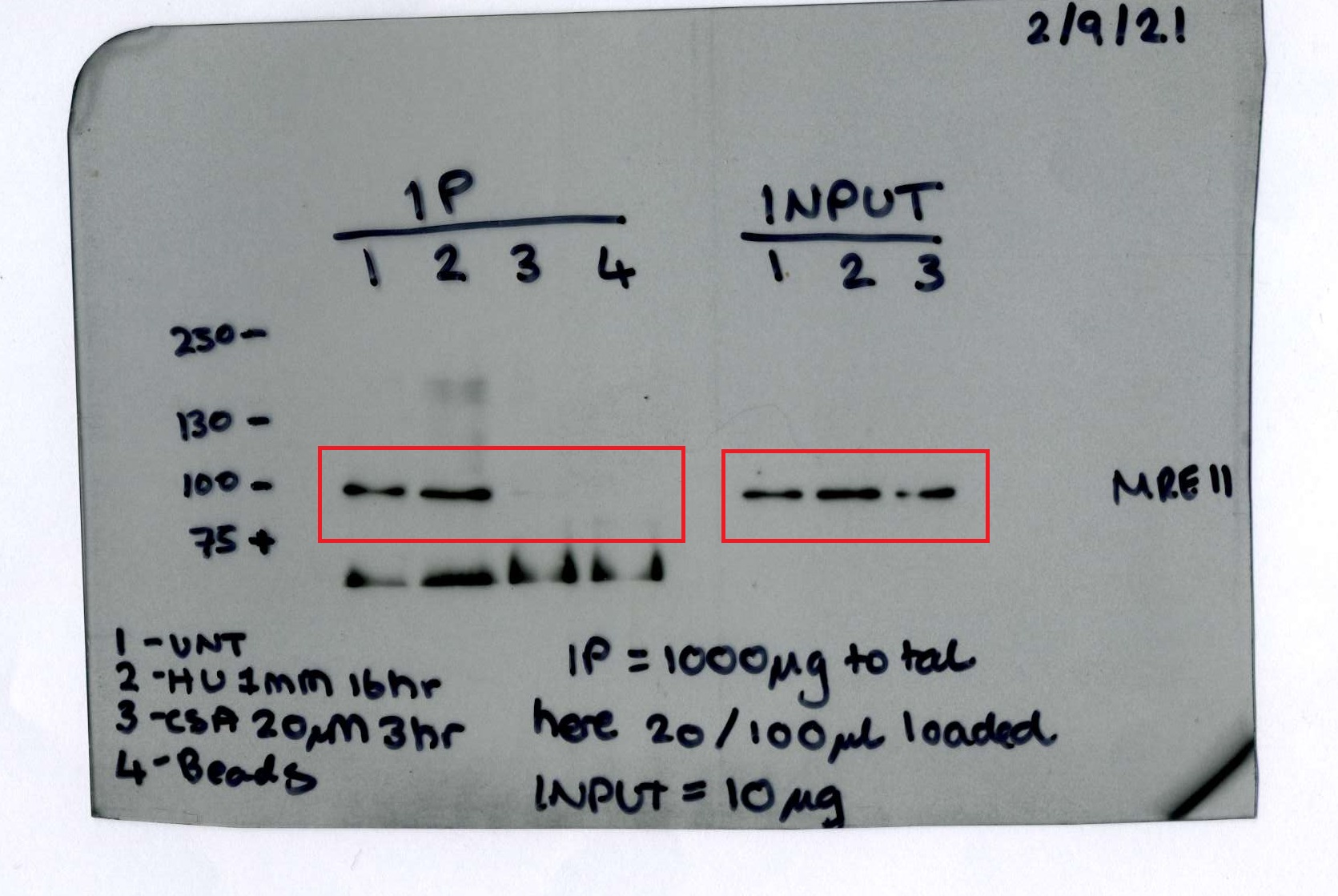

Supplement: Supplementary file 12 — Source data Fig. 7 [file 44319_2024_184_MOESM12_ESM.zip › Figure 7. Source Data/Fig 7A/Image data. Blot. MRE11 coIP.jpg]

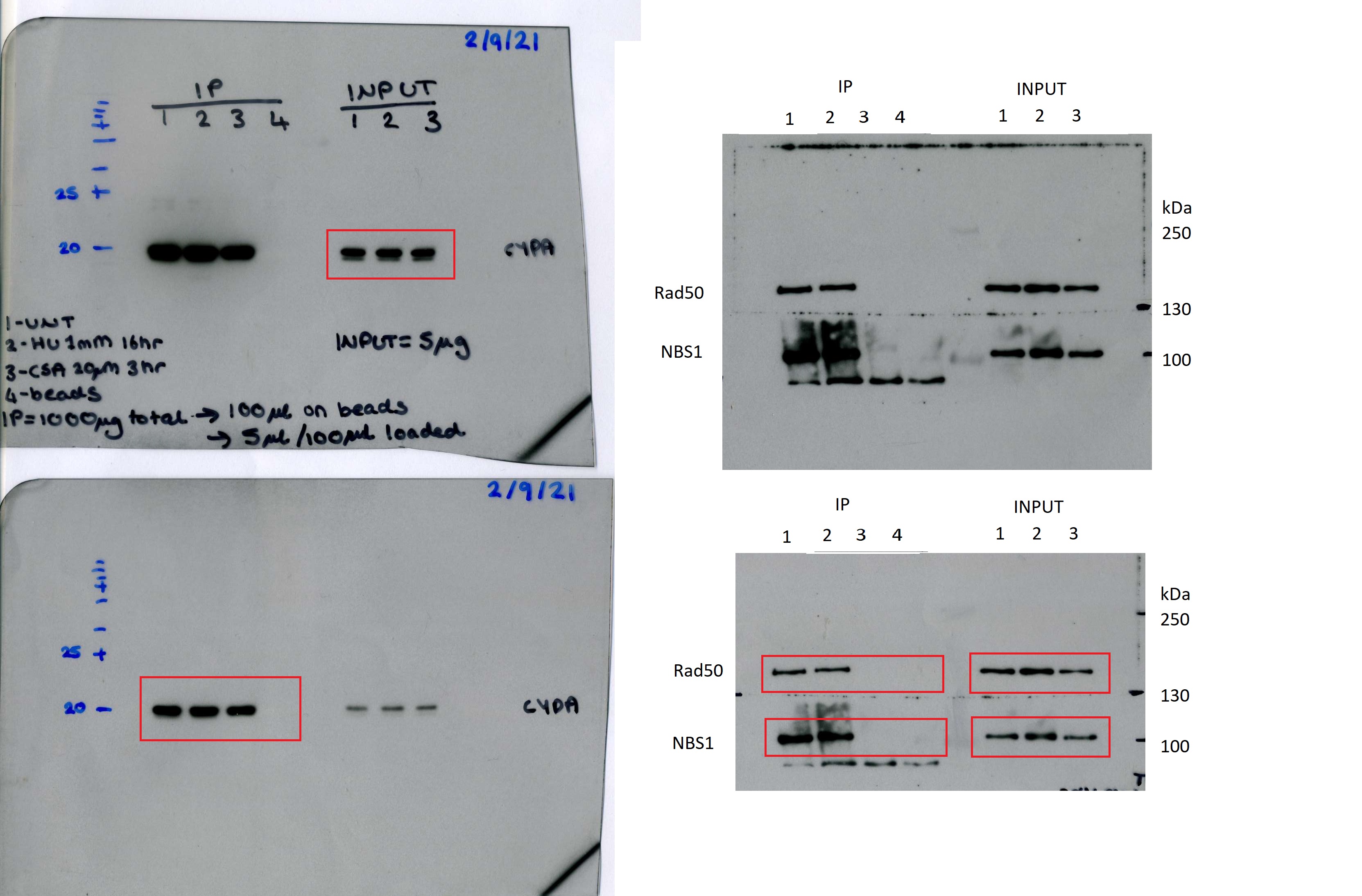

Supplement: Supplementary file 12 — Source data Fig. 7 [file 44319_2024_184_MOESM12_ESM.zip › Figure 7. Source Data/Fig 7A/Image data. Blot. NBS1, RAD50 coIP CYPA.jpg]

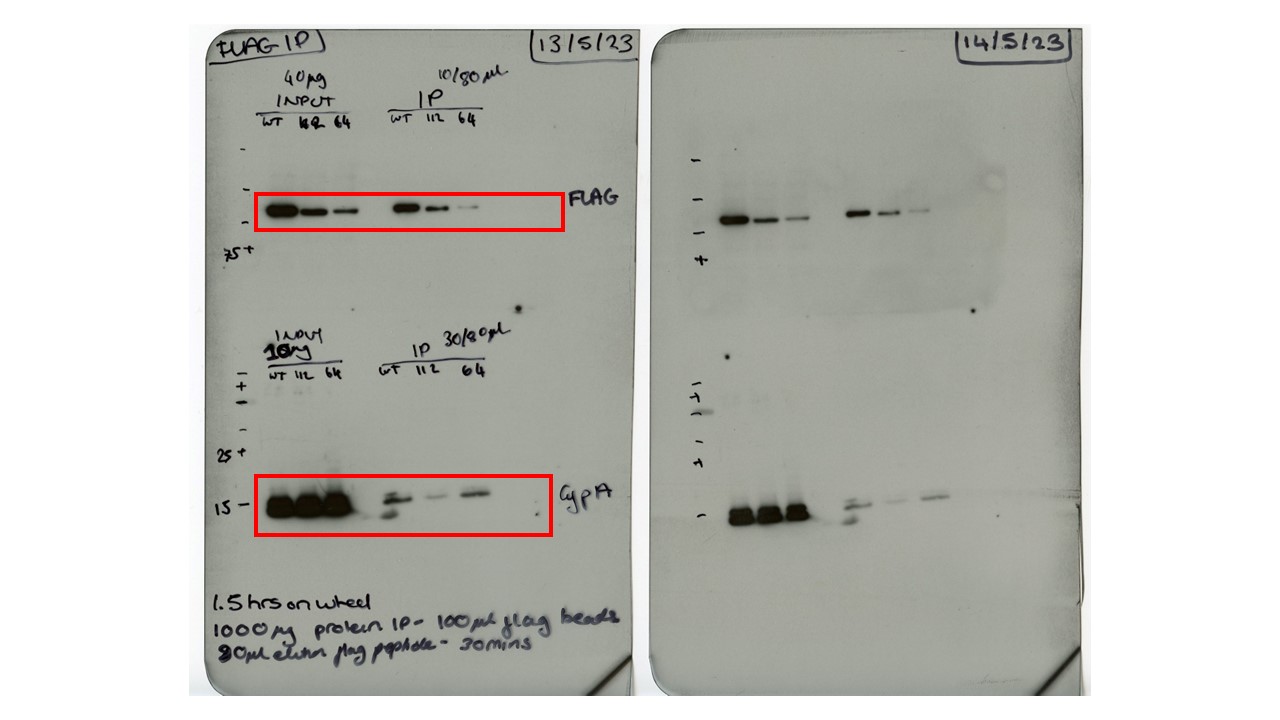

Supplement: Supplementary file 13 — Source data Fig. 8 [file 44319_2024_184_MOESM13_ESM.zip › Figure 8. Source Data/Fig 8A/Image data. Blot. FLAG and CYPA.jpg]

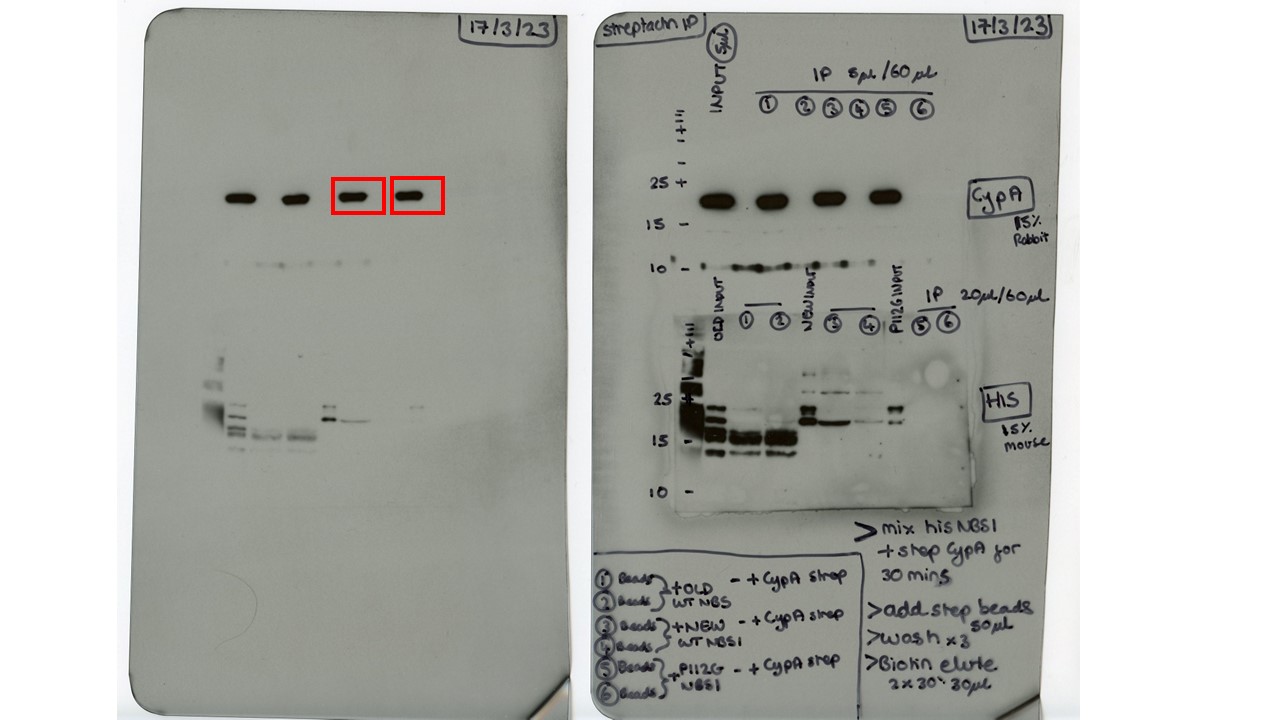

Supplement: Supplementary file 13 — Source data Fig. 8 [file 44319_2024_184_MOESM13_ESM.zip › Figure 8. Source Data/Fig 8B/Image data. Blot. CYPA.jpg]

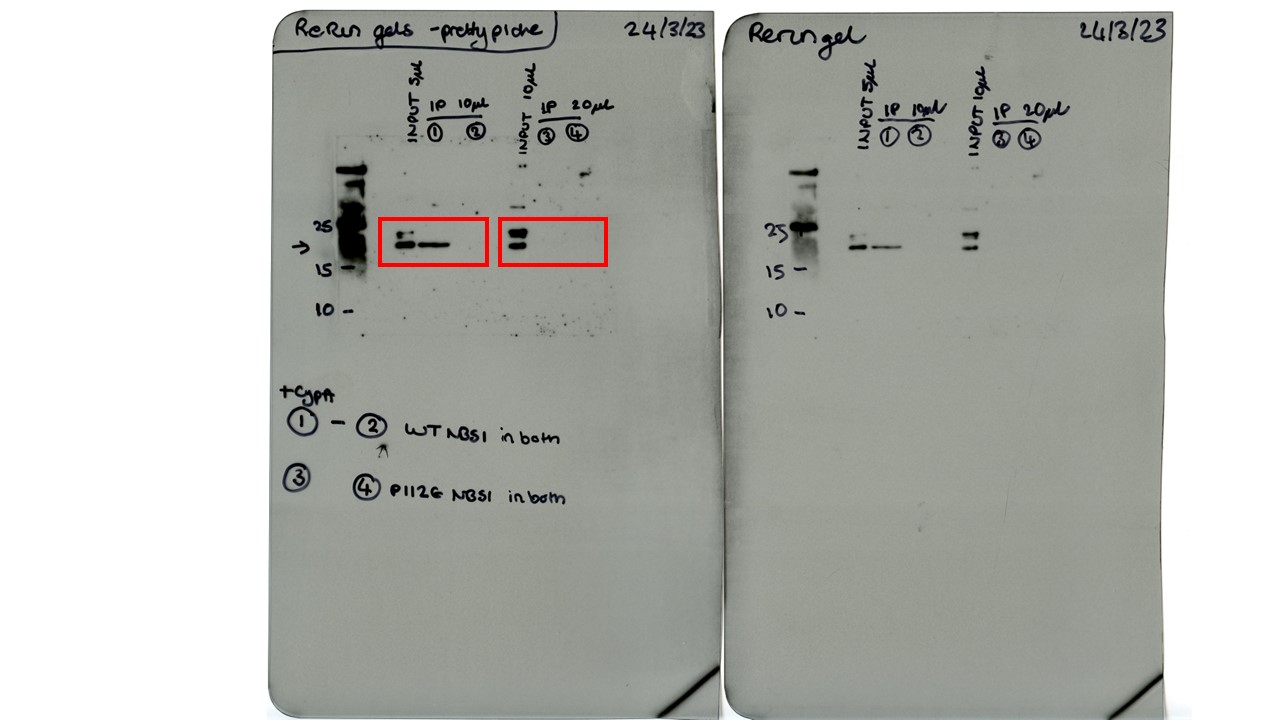

Supplement: Supplementary file 13 — Source data Fig. 8 [file 44319_2024_184_MOESM13_ESM.zip › Figure 8. Source Data/Fig 8B/Image data. Blot. HIS.jpg]

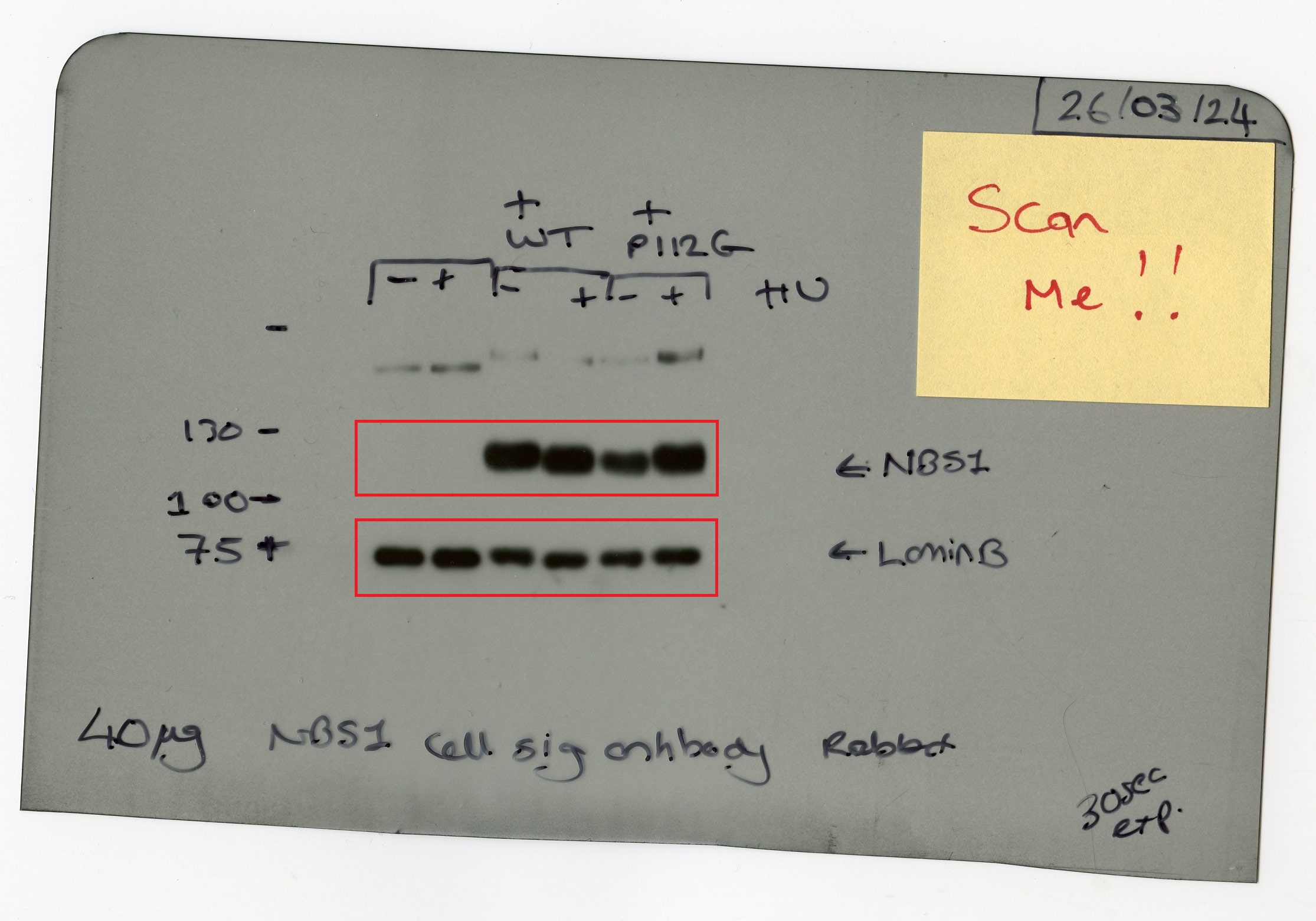

Supplement: Supplementary file 14 — Source data Fig. 9 [file 44319_2024_184_MOESM14_ESM.zip › Figure 9. Source Data/Fig 9C/Image data. Blot. NBS1 and Lamin B.jpg]

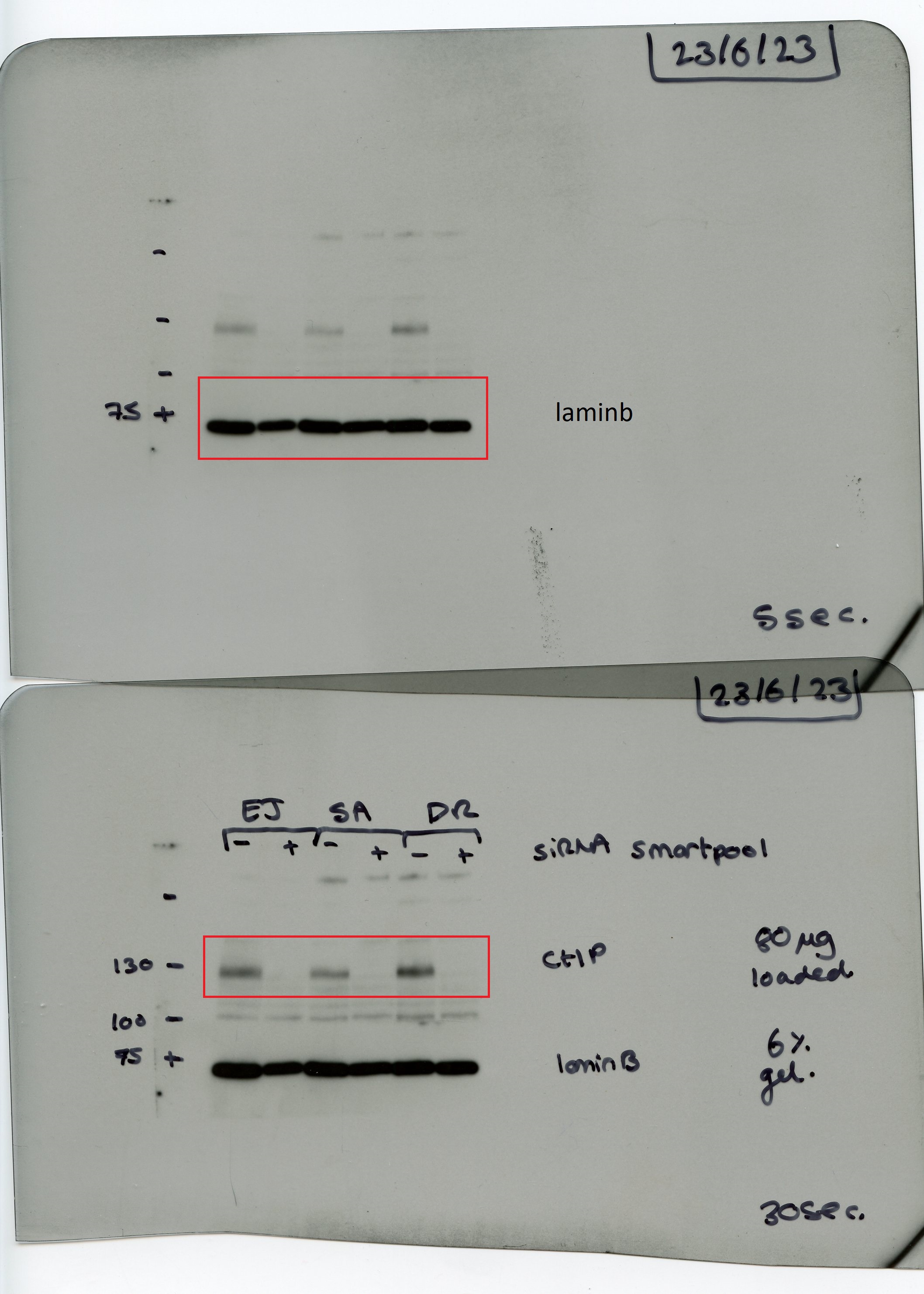

Supplement: Supplementary file 15 — Source data Fig. 10 [file 44319_2024_184_MOESM15_ESM.zip › Figure 10. Source Data/Fig 10A/Image data. Blot. CtIP and Lamin B.jpg]

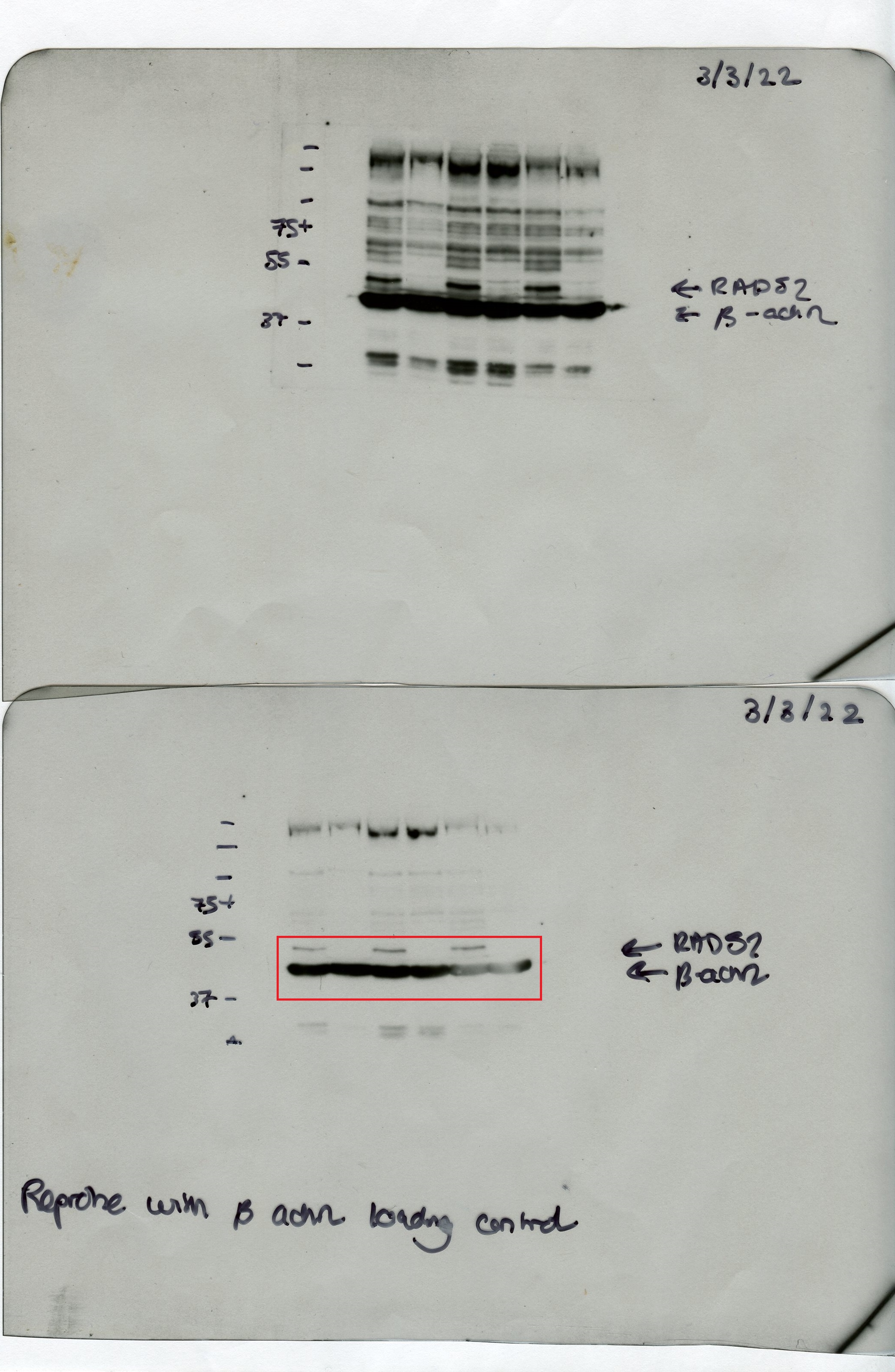

Supplement: Supplementary file 15 — Source data Fig. 10 [file 44319_2024_184_MOESM15_ESM.zip › Figure 10. Source Data/Fig 10B/Image data. Blot. beta actin.jpg]

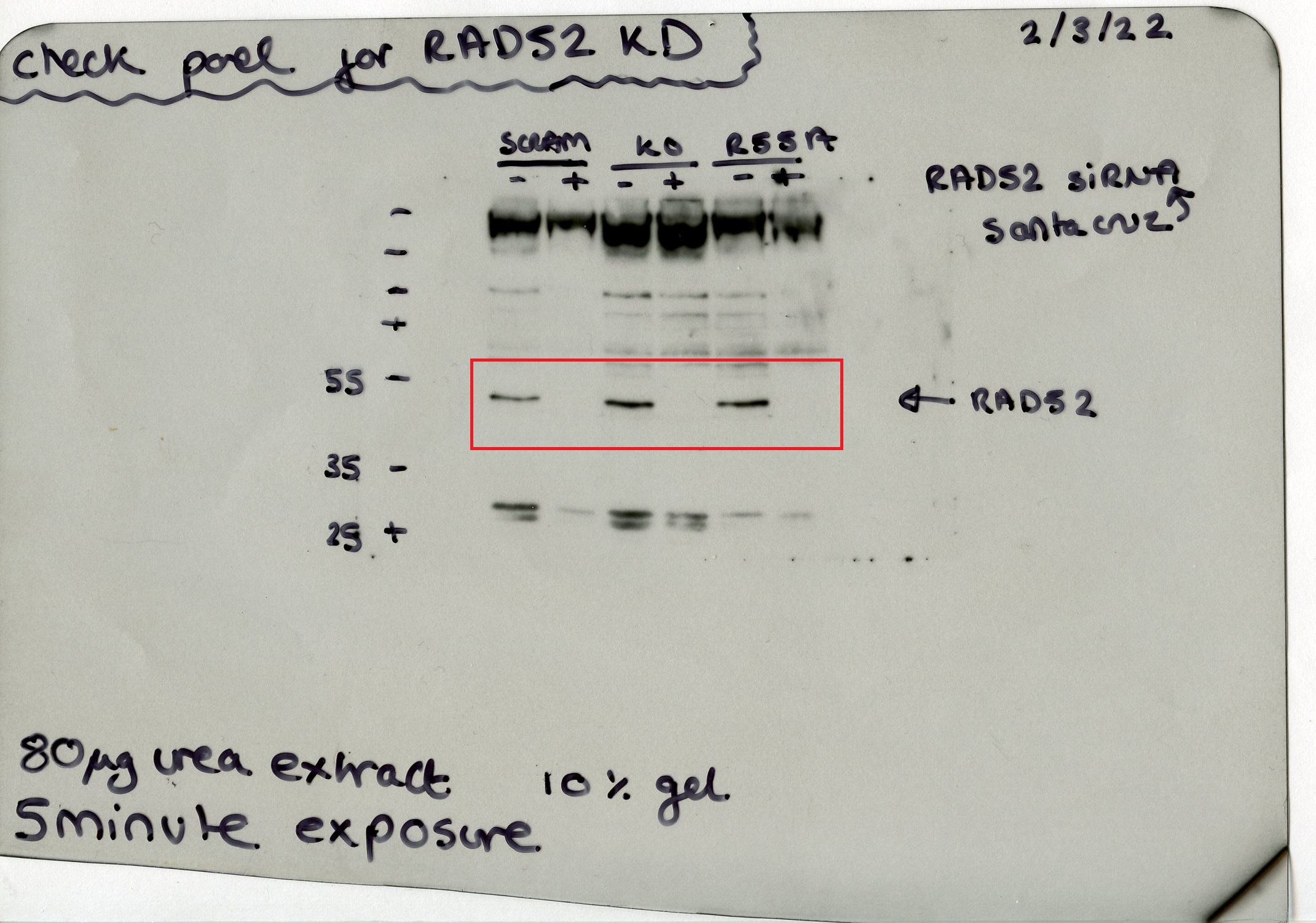

Supplement: Supplementary file 15 — Source data Fig. 10 [file 44319_2024_184_MOESM15_ESM.zip › Figure 10. Source Data/Fig 10B/Image data. Blot. RAD52.jpg]

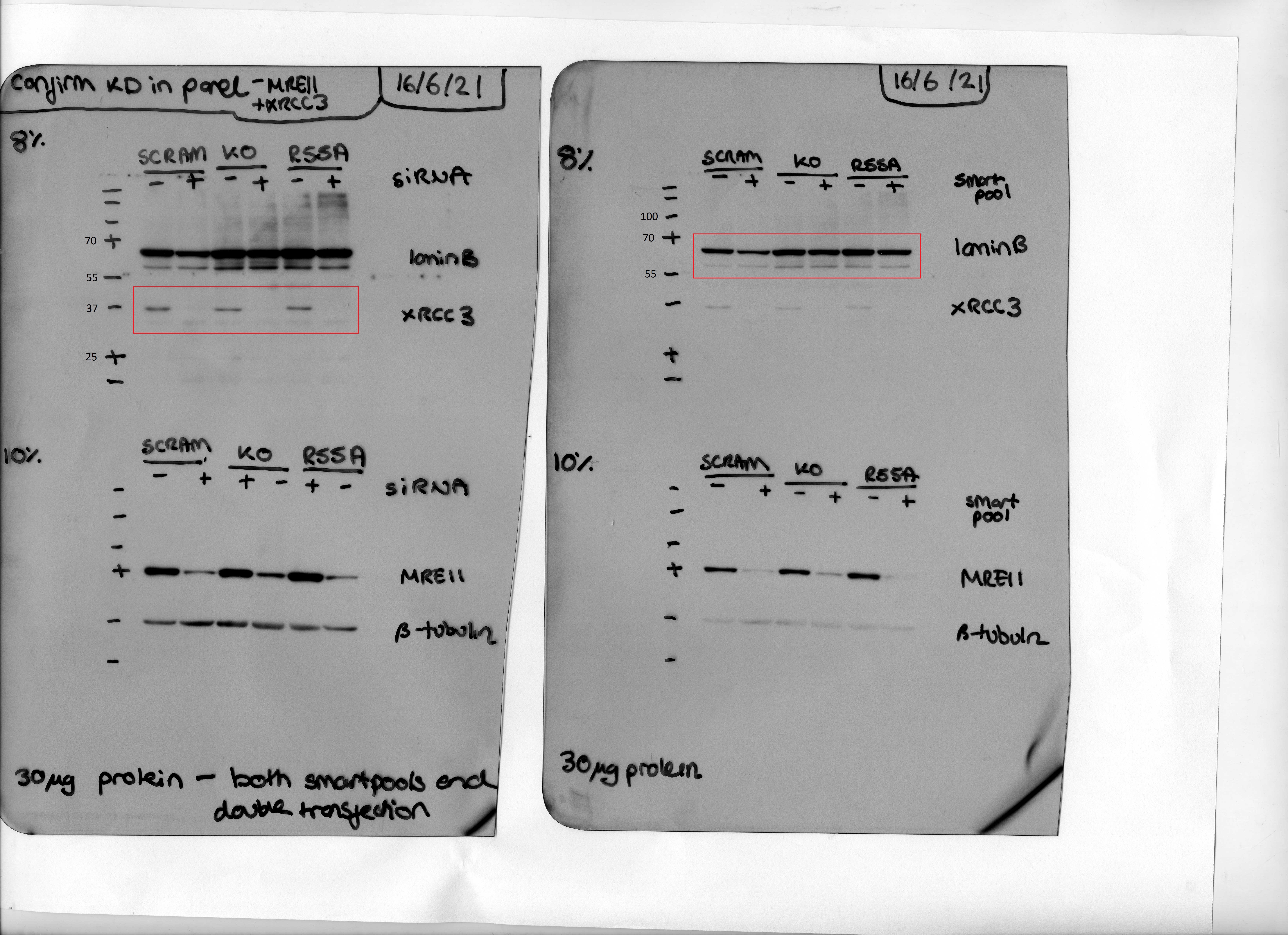

Supplement: Supplementary file 16 — Source data Fig. 11 [file 44319_2024_184_MOESM16_ESM.zip › Fig 11. SD/Fig 11C/Image data. Blot. XRCC3 and Lamin B.jpg]

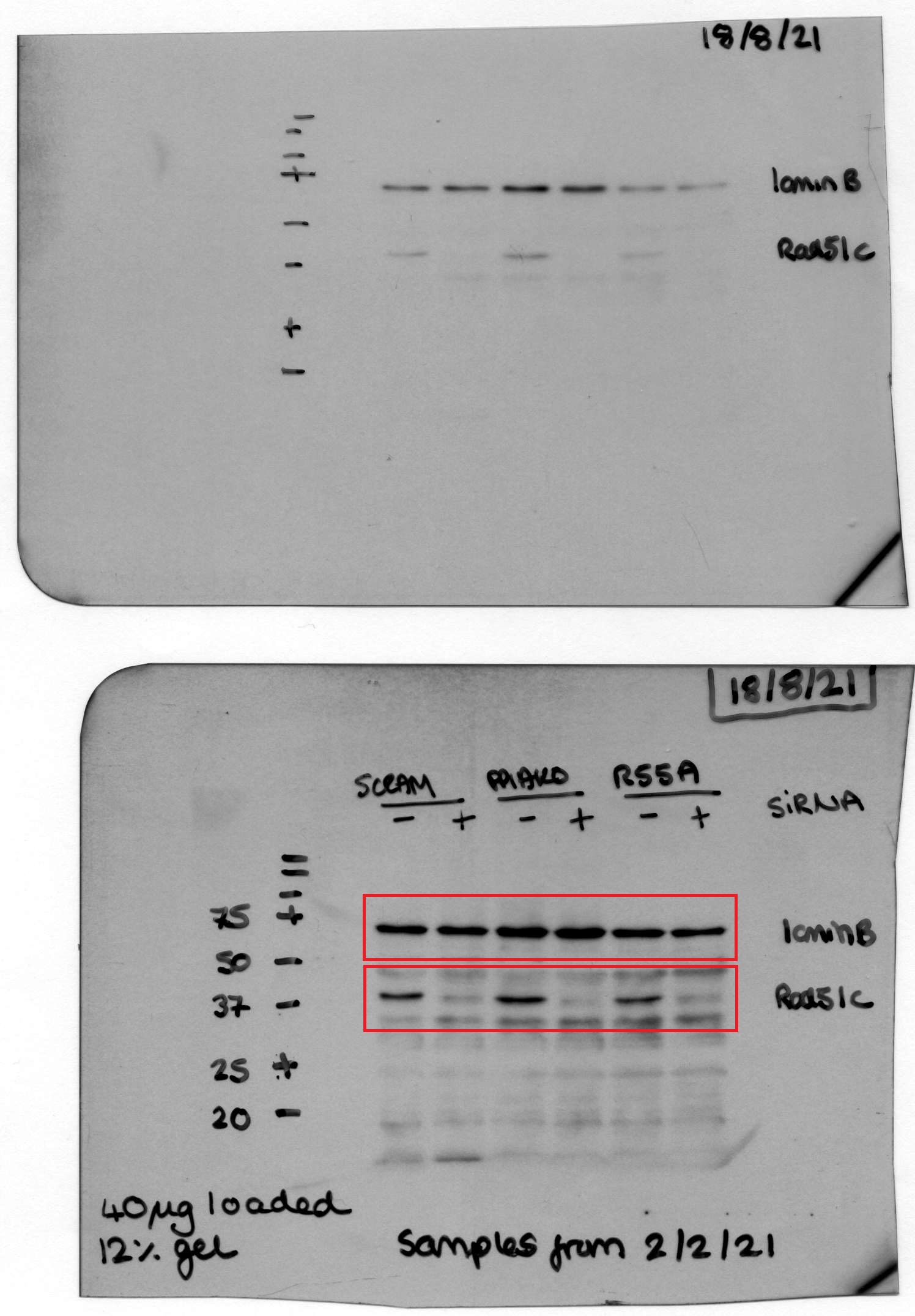

Supplement: Supplementary file 16 — Source data Fig. 11 [file 44319_2024_184_MOESM16_ESM.zip › Fig 11. SD/Fig 11D/Image data. Blot. RAD51C and Lamin B.jpg]

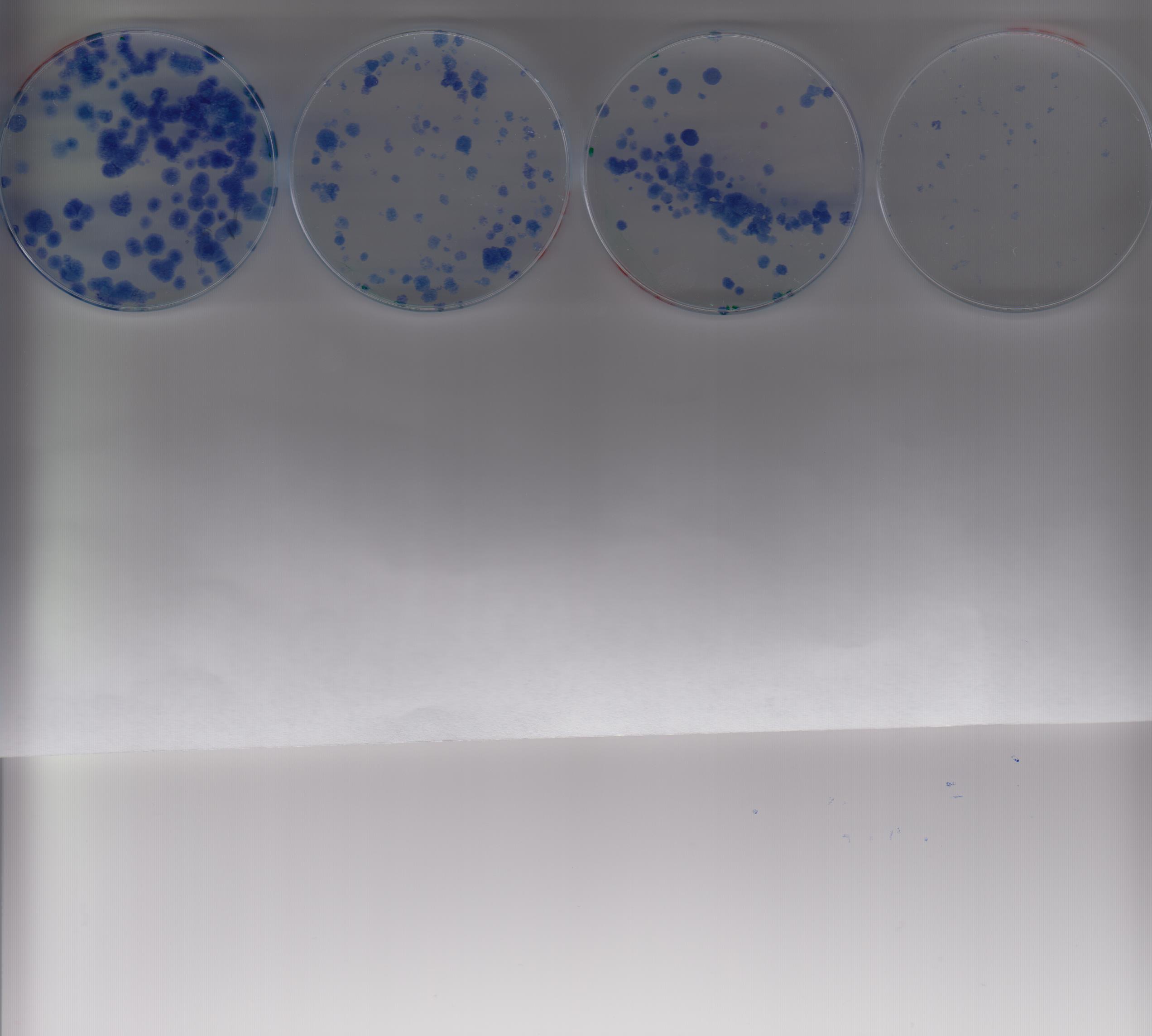

Supplement: Supplementary file 16 — Source data Fig. 11 [file 44319_2024_184_MOESM16_ESM.zip › Fig 11. SD/Fig 11E/shBRCA2 + and - CsA plate image..jpg]

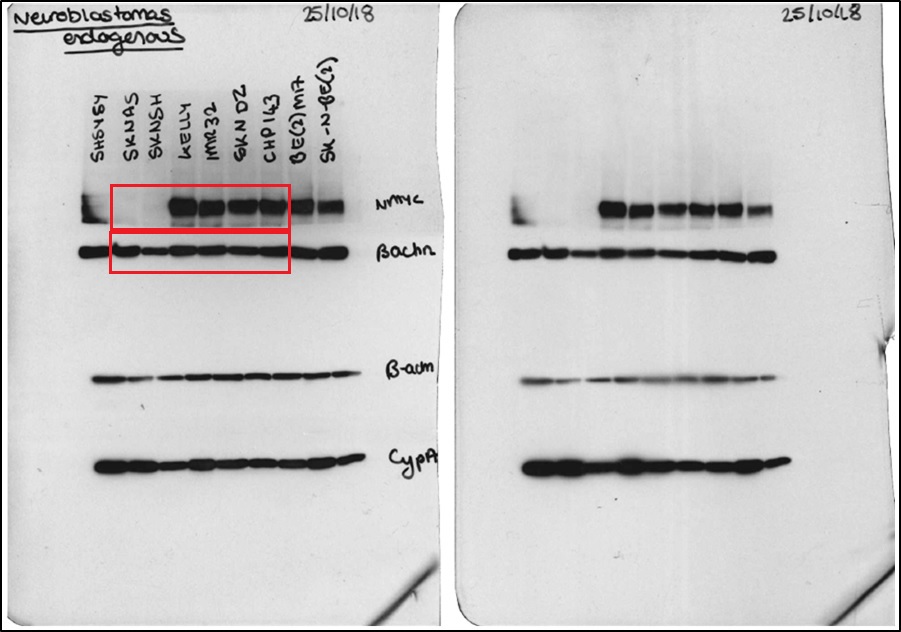

Supplement: Supplementary file 17 — Source data Fig. 12 [file 44319_2024_184_MOESM17_ESM.zip › Fig 12. SD/Fig 12A/Image data. Blot. MYCN and beta actin.jpg]

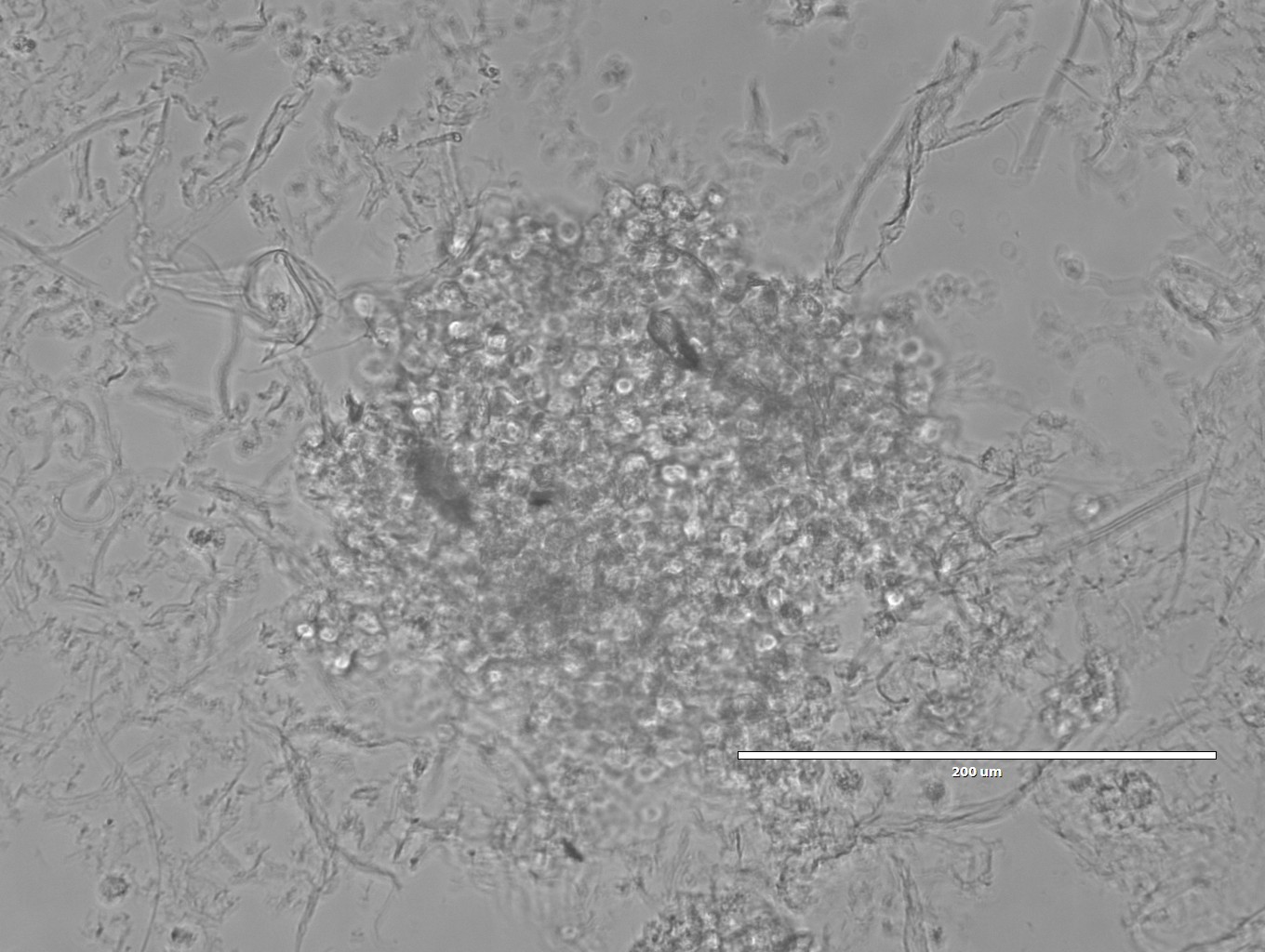

Supplement: Supplementary file 17 — Source data Fig. 12 [file 44319_2024_184_MOESM17_ESM.zip › Fig 12. SD/Fig 12C/KELLY. CsA. Micr.image.jpg]

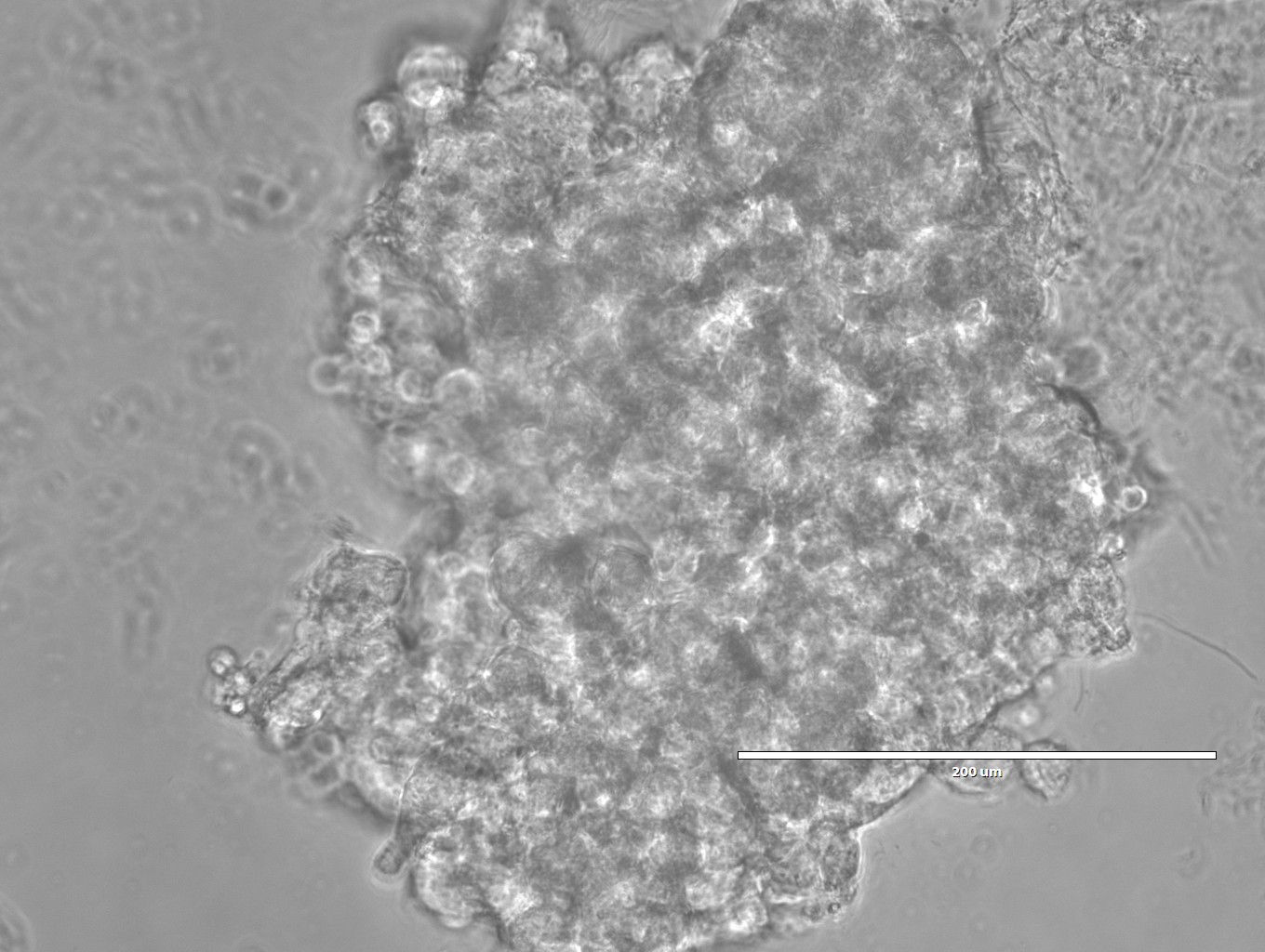

Supplement: Supplementary file 17 — Source data Fig. 12 [file 44319_2024_184_MOESM17_ESM.zip › Fig 12. SD/Fig 12C/KELLY. Unt. Micr.image.jpg]

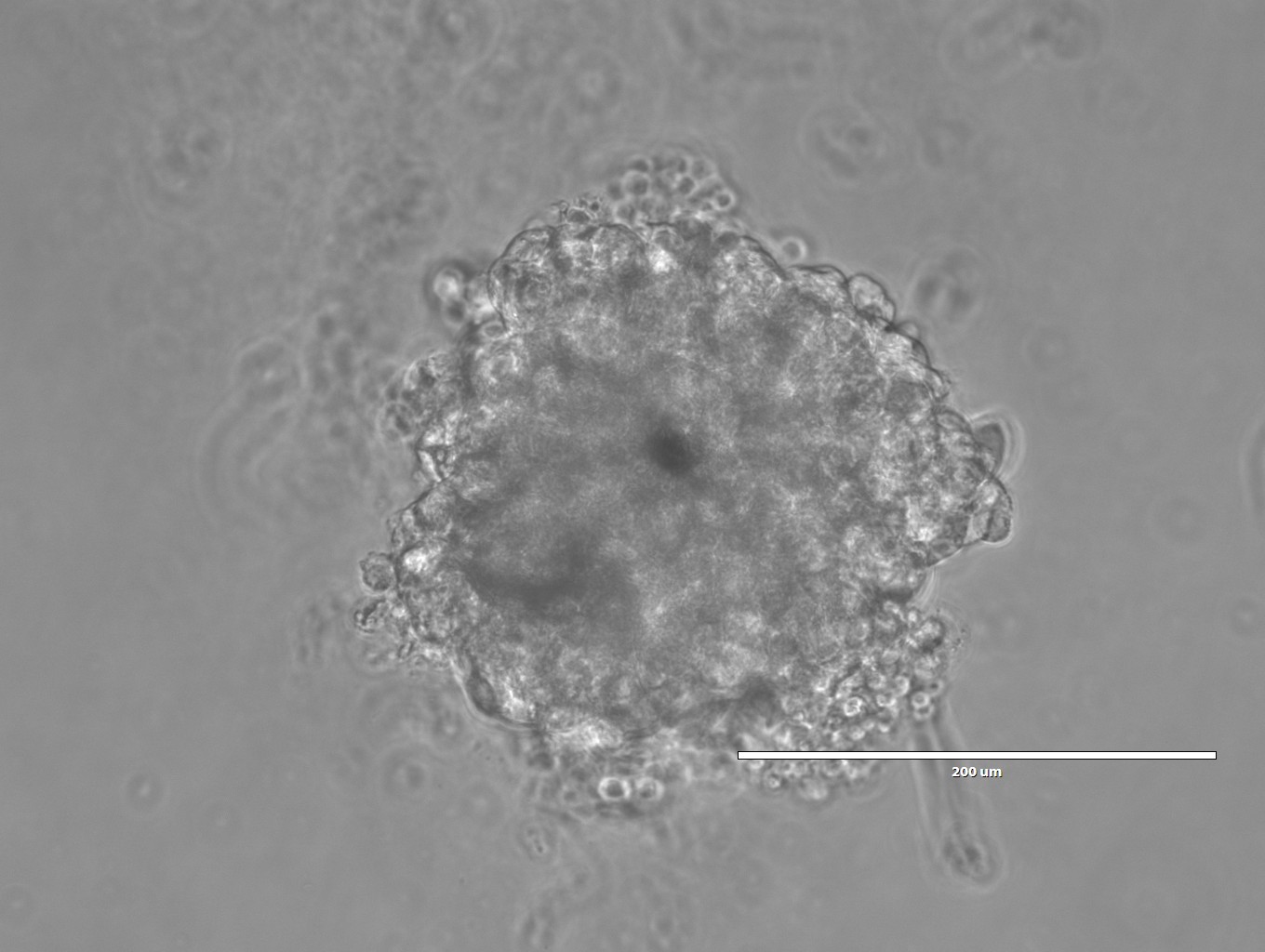

Supplement: Supplementary file 17 — Source data Fig. 12 [file 44319_2024_184_MOESM17_ESM.zip › Fig 12. SD/Fig 12C/SK-N-SH CsA. Micr.image.jpg]

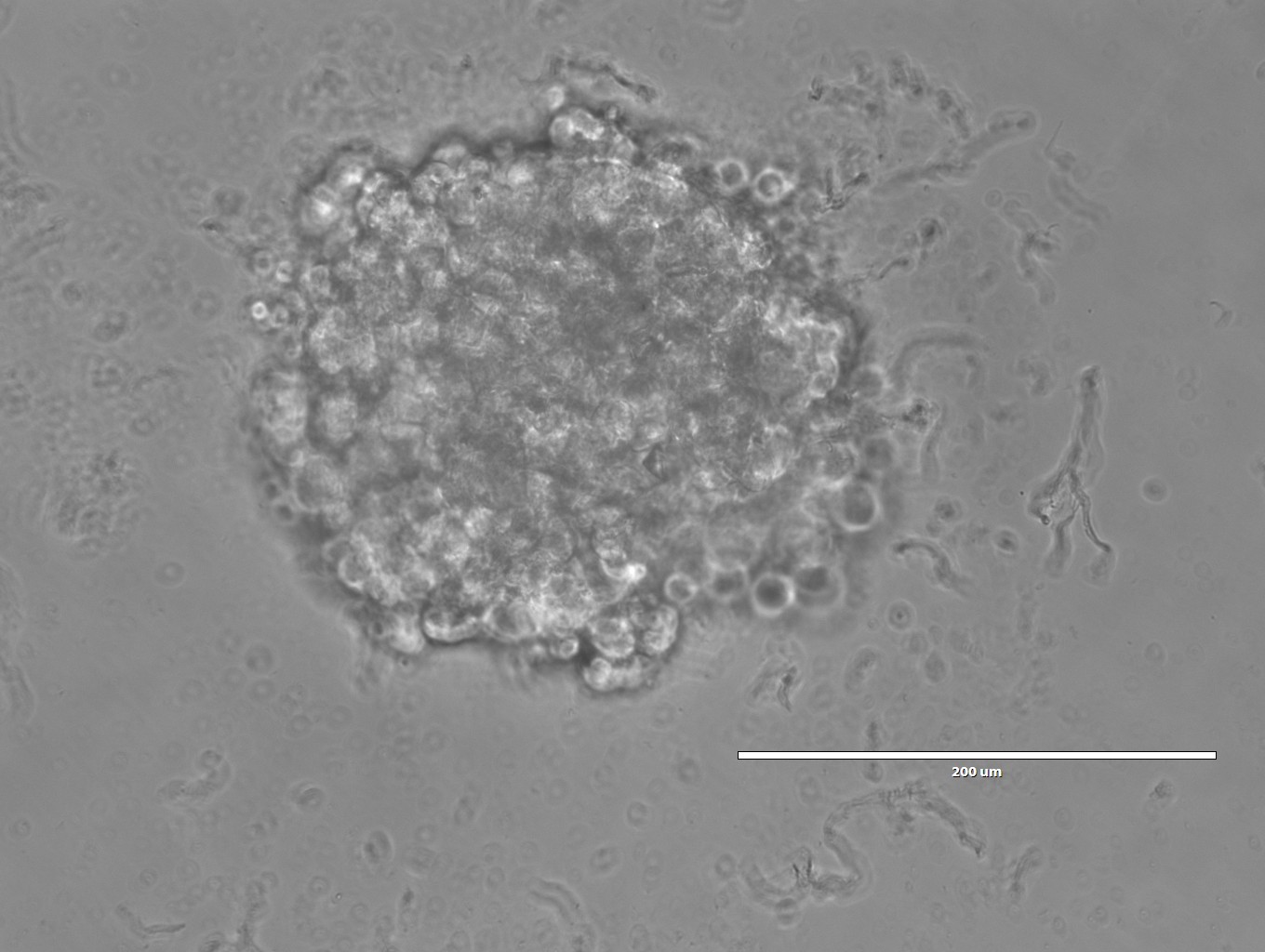

Supplement: Supplementary file 17 — Source data Fig. 12 [file 44319_2024_184_MOESM17_ESM.zip › Fig 12. SD/Fig 12C/SK-N-SH Unt. Micr.image.jpg]

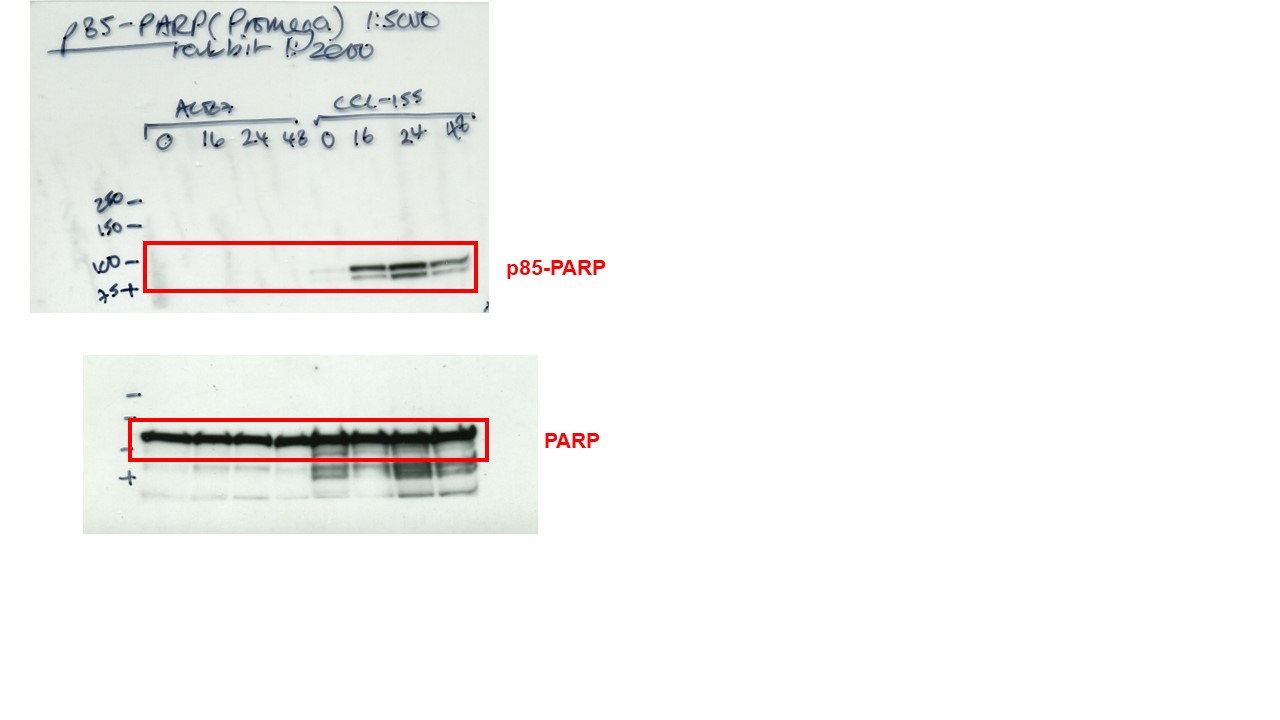

Supplement: Supplementary file 17 — Source data Fig. 12 [file 44319_2024_184_MOESM17_ESM.zip › Fig 12. SD/Fig 12E/Image data.. Blot. p85_PARP and PARP.jpg]

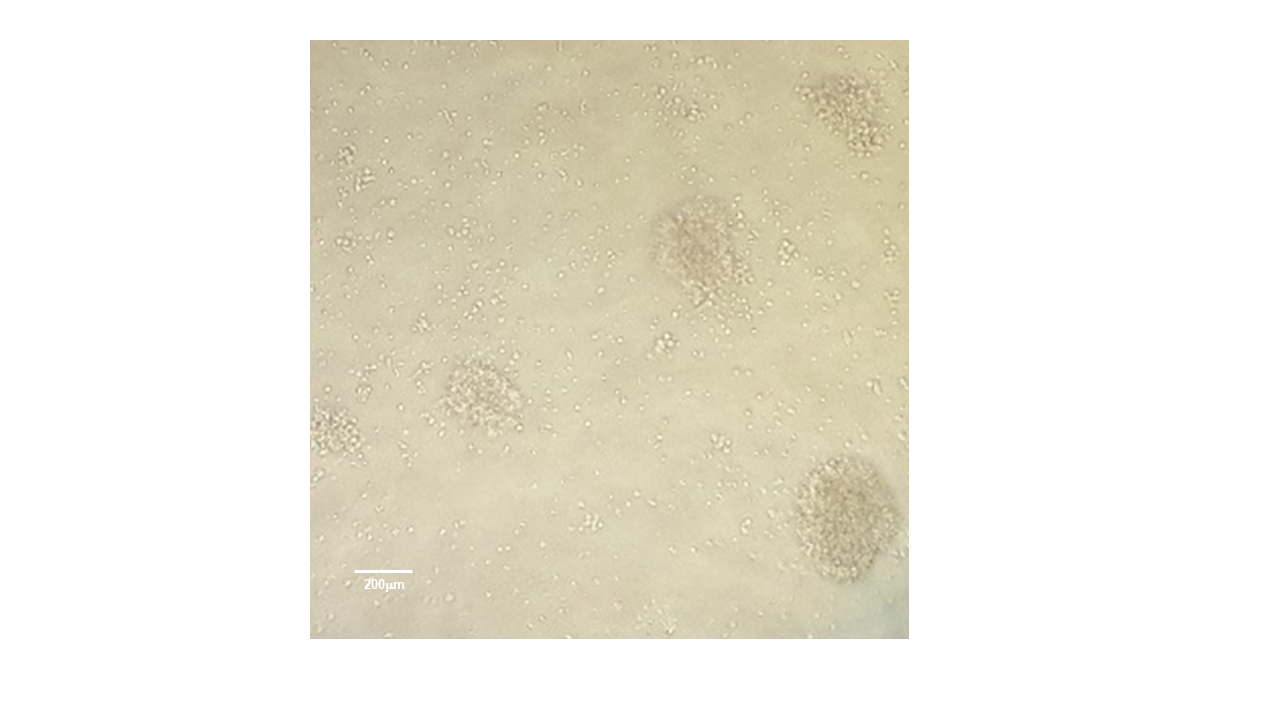

Supplement: Supplementary file 17 — Source data Fig. 12 [file 44319_2024_184_MOESM17_ESM.zip › Fig 12. SD/Fig 12F/3D Colonies RPMI-8226. Micr.image..tif]
