# Supplementary material for: A novel role for the peptidyl-prolyl cis-trans isomerase Cyclophilin A in DNA-repair following replication fork stalling via the MRE11-RAD50-NBS1 complex
Source: EMBO Rep. 2024 Jun 28;25(8):3432–55. doi: 10.1038/s44319-024-00184-9 (PMC11315929; doi:10.1038/s44319-024-00184-9)
Supplement: Supplementary file 11 — Source data Fig. 6 [file 44319_2024_184_MOESM11_ESM.zip › Figure 6. Source Data/Fig 6C/RAD51 Foci Box Plot Values. Numerical Data..pdf]

RAD51 Foci

Box plot statistics

	SCRAM UNT	SCRAM 3Hr, 2mM HU	SCRAM 16Hr, 1mM HU	PPIA KO UNT	PPIA KO 3Hr, 2mM HU	PPIA KO 16Hr, 1mM HU	R55A UNT	R55A 3Hr, 2mM HU	R55A 16Hr, 1mM HU
Upper whisker	28.00	65.00	41.00	24.00	16.00	29.00	7.00	11.00	19.00
3rd quartile	13.00	30.00	21.00	12.00	9.00	15.00	3.50	6.00	8.00
Median	7.00	12.50	15.00	6.50	2.00	5.00	2.00	1.00	4.00
1st quartile	3.00	3.00	7.00	2.00	0.00	3.00	1.00	0.00	0.00
Lower whisker	0.00	0.00	0.00	0.00	0.00	0.00	0.00	0.00	0.00
Nr. of data points	137.00	70.00	85.00	122.00	73.00	61.00	112.00	67.00	66.00
