# Supplementary material for: A novel role for the peptidyl-prolyl cis-trans isomerase Cyclophilin A in DNA-repair following replication fork stalling via the MRE11-RAD50-NBS1 complex
Source: EMBO Rep. 2024 Jun 28;25(8):3432–55. doi: 10.1038/s44319-024-00184-9 (PMC11315929; doi:10.1038/s44319-024-00184-9)
Supplement: Supplementary file 14 — Source data Fig. 9 [file 44319_2024_184_MOESM14_ESM.zip › Figure 9. Source Data/Fig 9A/NBS1 Foci Box Plot Values. Numerical Data..pdf]

NBS1 Foci

Box plot statistics

	SCRAM UNT	SCRAM 3Hr, 2mM HU	PPIA KO UNT	PPIA KO 3Hr, 2mM HU	R55A UNT	R55A 3Hr, 2mM HU
Upper whisker	29.00	40.00	33.00	41.00	40.00	29.00
3rd quartile	17.00	22.00	21.00	21.00	22.00	18.00
Median	12.00	15.00	15.00	15.00	15.50	12.00
1st quartile	8.50	10.00	11.00	7.00	10.00	9.00
Lower whisker	0.00	0.00	1.00	0.00	2.00	1.00
Nr. of data points	80.00	162.00	69.00	116.00	142.00	50.00
