# Supplementary material for: A novel role for the peptidyl-prolyl cis-trans isomerase Cyclophilin A in DNA-repair following replication fork stalling via the MRE11-RAD50-NBS1 complex
Source: EMBO Rep. 2024 Jun 28;25(8):3432–55. doi: 10.1038/s44319-024-00184-9 (PMC11315929; doi:10.1038/s44319-024-00184-9)
Supplement: Supplementary file 14 — Source data Fig. 9 [file 44319_2024_184_MOESM14_ESM.zip › Figure 9. Source Data/Fig 9B/MDC1 Foci Box Plot Values. Numerical Data..pdf]

MDC1 Foci

Box plot statistics

	SCRAM UNT	SCRAM 3Hr, 2mM HU	PPIA KO UNT	PPIA KO 3Hr, 2mM HU	R55A UNT	R55A 3Hr, 2mM HU
Upper whisker	54.00	90.00	84.00	88.00	76.00	84.00
3rd quartile	40.50	57.00	54.00	54.50	49.00	51.00
Median	33.00	42.00	42.00	41.00	38.00	39.50
1st quartile	27.00	28.00	31.00	31.00	29.00	29.00
Lower whisker	11.00	2.00	18.00	5.00	6.00	3.00
Nr. of data points	83.00	88.00	83.00	96.00	98.00	90.00
