# Supplementary material for: A novel role for the peptidyl-prolyl cis-trans isomerase Cyclophilin A in DNA-repair following replication fork stalling via the MRE11-RAD50-NBS1 complex
Source: EMBO Rep. 2024 Jun 28;25(8):3432–55. doi: 10.1038/s44319-024-00184-9 (PMC11315929; doi:10.1038/s44319-024-00184-9)
Supplement: Supplementary file 14 — Source data Fig. 9 [file 44319_2024_184_MOESM14_ESM.zip › Figure 9. Source Data/Fig 9D/NBS1 Foci Box Plot Values. Numerical Data..pdf]

Box scatter plot statistics **NBS-ILB1** Nijmegen breakage syndrome patient-derived FBs.

Cellosaurus accession no. CVCL_XF97

Originally described in Pubmed id: 10377945. Kraakman-van der Zwer M *et al* Mut Res 1999. Obtained from Dr. Malgorzata Z Zdzienicka, MGC-Department of Radiation Genetics and Chemical Mutagenesis, Leiden University-LUMC, Netherlands

SV40 transformed skin fibroblasts homozygous for the Nijmegen breakage syndrome *NBN* founder mutation: (657del5), p.Lys219Asn*fs**16 (c.657_661delACAAA)

HU-induced NBS1 foci.

WT Unt *Vs* HU t-test: *2-tail, unequal variance*
$p = 1.49 \times 10^{-5}$

P112G Unt *Vs* HU t-test: 2-tail, unequal variance
p = 0.622703

Median values:

WT Unt:	26	WT HU:	39
P112G Unt:	27	P112G HU:	28

Mean values:

WT Unt:	32.52	WT HU:	54.27
P112G Unt:	34.94	P112G HU:	38.42

No of datapoints:

WT Unt:	64	WT HU:	111
P112G Unt:	53	P112G HU:	33
